# Transcriptome analysis of early stages of sorghum grain mold disease reveals defense regulators and metabolic pathways associated with resistance

**DOI:** 10.1186/s12864-021-07609-y

**Published:** 2021-04-22

**Authors:** Habte Nida, Sanghun Lee, Ying Li, Tesfaye Mengiste

**Affiliations:** 1 Department of Botany and Plant Pathology, Purdue University, West Lafayette, IN 47907 USA; 2 Department of Horticulture and Landscape Architecture, Purdue University, West Lafayette, IN 47907 USA

**Keywords:** Sorghum, Grain mold, Grain transcriptome, Mold fungi

## Abstract

**Background:**

Sorghum grain mold is the most important disease of the crop. The disease results from simultaneous infection of the grain by multiple fungal species. Host responses to these fungi and the underlying molecular and cellular processes are poorly understood. To understand the genetic, molecular and biochemical components of grain mold resistance, transcriptome profiles of the developing grain of resistant and susceptible sorghum genotypes were studied.

**Results:**

The developing kernels of grain mold resistant RTx2911 and susceptible RTx430 sorghum genotypes were inoculated with a mixture of fungal pathogens mimicking the species complexity of the disease under natural infestation. Global transcriptome changes corresponding to multiple molecular and cellular processes, and biological functions including defense, secondary metabolism, and flavonoid biosynthesis were observed with differential regulation in the two genotypes. Genes encoding pattern recognition receptors (PRRs), regulators of growth and defense homeostasis, antimicrobial peptides, pathogenesis-related proteins, zein seed storage proteins, and phytoalexins showed increased expression correlating with resistance. Notably, *SbLYK5* gene encoding an orthologue of chitin PRR, defensin genes *SbDFN7.1* and *SbDFN7.2* exhibited higher expression in the resistant genotype. The *SbDFN7.1* and *SbDFN7.2* genes are tightly linked and transcribed in opposite orientation with a likely common bidirectional promoter. Interestingly, increased expression of JAZ and other transcriptional repressors were observed that suggested the tight regulation of plant defense and growth. The data suggest a pathogen inducible defense system in the developing grain of sorghum that involves the chitin PRR, MAPKs, key transcription factors, downstream components regulating immune gene expression and accumulation of defense molecules. We propose a model through which the biosynthesis of 3-deoxyanthocynidin phytoalexins, defensins, PR proteins, other antimicrobial peptides, and defense suppressing proteins are regulated by a pathogen inducible defense system in the developing grain.

**Conclusions:**

The transcriptome data from a rarely studied tissue shed light into genetic, molecular, and biochemical components of disease resistance and suggested that the developing grain shares conserved immune response mechanisms but also components uniquely enriched in the grain. Resistance was associated with increased expression of genes encoding regulatory factors, novel grain specific antimicrobial peptides including defensins and storage proteins that are potential targets for crop improvement.

**Supplementary Information:**

The online version contains supplementary material available at 10.1186/s12864-021-07609-y.

## Background

Sorghum [*Sorghum bicolor* (L.) Moench] is among the world’s most important cereal crops used for food, feed, and bio-fuels with unique adaptation to arid and semi-arid parts of the world. Grain mold is the most important and complex disease of sorghum caused by different pathogenic fungal species mainly in the genus *Fusarium*, but also including species in the genera *Curvularia*, *Alternaria*, *Phoma*, *Bipolaris*, *Exserohilum*, *Aspergillus*, *Colletotrichum*, and *Penicillium*. Grain mold is widespread, with major impacts on grain yield and quality especially in regions with high humidity during grain development and harvest with highly detrimental effects on grain quality due to contamination by mycotoxins. The closely related diseases include the *Fusarium* ear rot of corn and *Fusarium* head blight of wheat, which are all caused by similar group of fungal pathogens with necrotrophic mode of nutrition.

Prior studies conducted on sorghum indicate that resistance to grain mold is associated with grain flavonoids such as testa pigmentation, concentration of phenolic compounds, 3-deoxyanthocynidns, tannins and grain physical characteristics such as grain hardness [1–4]. These observations are mainly based on trait correlations but the underlying genetics of grain mold resistance remained unclear. Recent advances in sequencing technologies, substantial reduction in the cost of genotyping and availability of efficient bioinformatics tools brought new opportunities to determine the genetic control of complex phenotypes at greater depth. Global transcriptome profiling enables the identification of genome wide variations in gene expression associated with traits of interest. Transcriptional control of gene expression is a widespread regulatory event in plant responses to pathogen infection. This is particularly important since many genes associated with disease resistance are known to be transcriptionally regulated, and such an approach may identify genes mediating responses to pathogens, with a subset likely having direct contribution to resistance. Despite numerous transcriptome studies conducted in response to pathogen infection in leaf tissue, the transcriptome responses of the grain to pathogen attack have not been studied. Consequently, the processes and pathways activated or repressed during infection remain poorly understood.

Here, we conducted a comparative transcriptome analysis of grain mold resistant and susceptible sorghum genotypes RTx2911 and RTx430, respectively. The transcriptome profiling was conducted on RNA samples from developing grain (20 days after flowering) inoculated with a combination of fungal species known to constitute the grain mold fungal complex in sorghum. Subsequently, we found differential expression of regulatory genes, signaling components associated with major immune response pathways and potential defense active molecules. Key defense mechanisms activated in the resistant genotype in response to infection were identified providing new understandings about the genetic and molecular bases of resistance to grain mold. A subset of these define novel defense strategies against fungal infection that are likely to be specific to grain tissues. Genetic and molecular dissection of defense responses in grain presents unique challenges, and our study lays the foundation for further genetic studies in grain mold resistance of sorghum.

## Results

### RNA sequence data from resistant and susceptible genotypes and mapping to the BTx623 and RTx430 reference genomes

A total of 433,396,806 raw reads and 432,025,256 adapter trimmed and quality clipped reads were generated for the 12 RNA-seq libraries (Table 1). Each sample was represented by an average of 36 million high quality reads. The adaptor trimmed and quality clipped reads were mapped to the *Sorghum bicolor* reference genomes [5, 6] with 75 to 84% of the reads uniquely aligned to the reference genomes (BTx623 and RTx430) in each sample.

**Table 1:** Summary statistics of RNA-seq reads generated through the HiSeq 2500 ultra-high-throughput sequencing system

Genotype	Time point	Raw reads combined together		Adapter trimmed & Quality clipped reads		Mapping to reference genomes (%)	
		Total reads	Bases	Total reads	Bases	BTx623	RTx430
RTx2911	0 h	35,193,900	5,314,278,900	35,029,678	5,133,585,627	82.50	83.68
RTx2911	0 h	34,912,900	5,271,847,900	34,631,646	4,913,623,155	82.73	82.34
RTx2911	0 h	33,077,984	4,994,775,584	32,974,808	4,785,724,826	71.82	74.36
RTx2911	24 h	38,532,904	5,818,468,504	38,395,698	5,647,473,903	77.70	80.06
RTx2911	24 h	52,634,848	7,947,862,048	52,565,274	7,712,587,392	78.28	79.00
RTx2911	24 h	46,206,820	6,977,229,820	46,082,874	6,688,093,720	74.55	75.34
RTx430	0 h	40,628,242	6,134,864,542	40,426,498	5,894,899,883	76.98	80.88
RTx430	0 h	33,352,520	5,036,230,520	33,298,302	4,868,287,180	75.33	79.36
RTx430	0 h	23,129,326	3,492,528,226	23,079,728	3,369,245,420	77.87	81.21
RTx430	24 h	28,798,260	4,348,537,260	28,741,444	4,245,933,800	77.35	80.66
RTx430	24 h	33,903,182	5,119,380,482	33,844,156	4,979,185,158	77.93	81.57
RTx430	24 h	33,025,920	4,986,913,920	32,955,150	4,855,366,073	78.40	82.01

### Overview of differential gene expression in healthy and inoculated developing grain

**Fig. 1:**
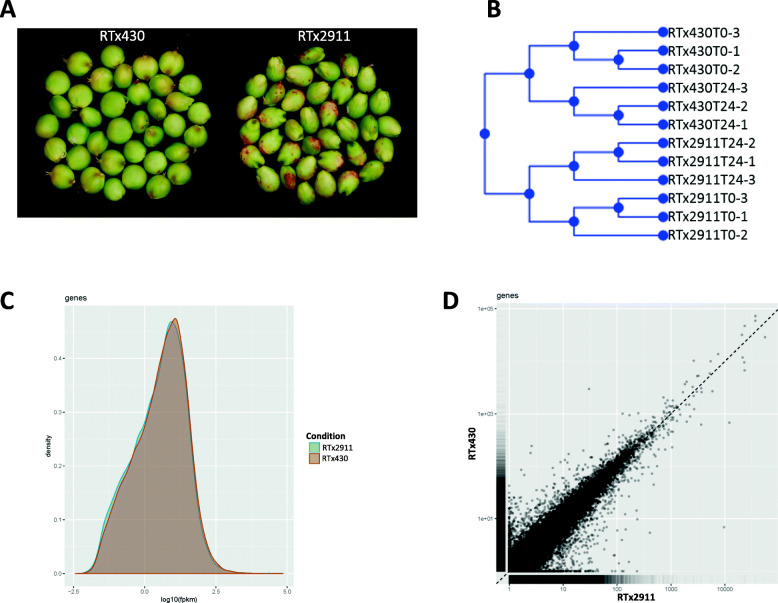
Developing grain of sorghum used for transcriptome analyses. **a** Grain of sorghum RTx430 and RTx2911 at 20 days after flowering used for total RNA extraction. **b** Hierarchical clustering of samples based on Euclidean distances. **c** CummeRbund plots of the expression level distribution for all genes in RTx2911 and RTx430 at 24 h after inoculation. FPKM, fragments per kilobase of transcript per million fragments mapped. **d** CummeRbund scatter plots highlighting general similarities and specific outliers between RTx2911 and RTx430 at 24 h after inoculation

To identify differentially expressed genes related to grain mold resistance, transcriptomes were compared between the resistant (RTx2911) and susceptible (RTx430) genotypes. Developing grains of the two genotypes (Fig. 1a) were inoculated with conidial suspension from a consortium of *Fusarium* and *Alternaria* species and sampled at 0 and 24 h post inoculation (hpi) for RNA extraction which was subsequently used for RNA-seq. Hierarchical clustering analysis of expression data of all samples indicated distinct clustering by genotype, RTx2911 and RTx430, and pathogen inoculation (Fig. 1b). CummeRbund plots of expression level distribution (Fig. 1c) indicated a typical expression profile while the scatter plot (Fig. 1d) highlighted the overall similarities and outliers between the two genotypes. Transcriptome comparisons were made between the two genotypes at each time point and within each of the genotypes at the two time points.

### Genotype dependent differential gene expression

**Fig. 2:**
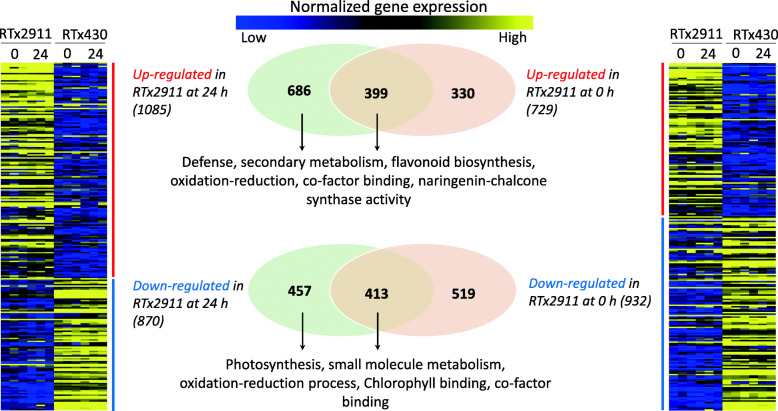
Genes differentially expressed between grain mold resistant and susceptible genotypes and significantly enriched gene ontology (GO) terms. GO terms displayed indicate those with highest significance

Comparisons of gene expression profiles between genotypes identified a number of genes that were differentially expressed between RTx2911 and RTx430 (Fig. 2). A total of 1661 genes were differentially expressed at 0 hpi, of which 729 showed higher expression and 932 showed lower expression in RTx2911 compared to RTx430 (Table S1). At 24 hpi, 1955 genes were differentially expressed, of which 1085 were up-regulated and 870 were down-regulated in RTx2911 compared to RTx430 (Table S2). Some of these up and down-regulated genes were common between 0 and 24 hpi. These include 399 up-regulated and 413 down-regulated genes in RTx2911 compared to RTx430.

Gene ontology (GO) enrichment analysis of genes differentially expressed between RTx2911 and RTx430 at 0 hpi revealed that there were no significantly enriched GO terms associated with this time point whereas analysis of genes differentially expressed at 24 hpi resulted in significantly enriched GO biological, molecular, kyoto encyclopedia of genes and genomes (KEGG) [7] and cellular terms. The complete list of genes under each of these GO terms indicating processes with highly significant enrichment are presented at the top and least significant once at bottom (Fig. S1-S3).

### Gene ontology analyses of biological process regulated by fungal infection in the grain

GO enrichment analysis of genes up-regulated in the resistant genotype RTx2911 at 24 hpi identified biological processes that include defense response with 31 genes, biosynthesis of secondary metabolites with 22 genes, flavonoid biosynthesis with 6 genes, and oxidation-reduction with 87 genes (Fig. 2, Fig. S1a). On the other hand, significantly enriched biological processes that were down-regulated in RTx2911 at 24 hpi included photosynthesis with 32 genes, small molecule metabolic process with 60 genes, and oxidation-reduction process with 79 genes (Fig. 2, Fig. S1b).

The 31 defense response genes that were up-regulated in RTx2911 at 24 hpi include pathogenesis-related (PR) genes, NPR1/NIM1 (Sobic.001G143000), NPR1 interacting (Sobic.003G086200), defensins (gamma-thionin), antimicrobial peptides, receptor like kinases, WRKY transcription factors, jasmonate ZIM domain (JAZ), isoflavone 2′-hydroxylase, CHY zinc finger, and a putative nematode-resistance gene (Table 2). PR genes are widely known as markers of immune response activation and contribute to defense pathways including systemic acquired resistance [8] while the NPR1gene is required for both systemic acquired resistance and induced systemic resistance [9, 10]. The Sobic.004G317500 gene that encodes norcoclaurine synthase which is a member of PR10 related protein family was also up-regulated in RTx2911 [11]. Defensins (formerly called gamma-thionin) are small, highly stable, cysteine-rich antimicrobial peptides which are components of the plant immune response [12, 13]. Six defensin genes were up-regulated in response to inoculation in the resistant genotype RTx2911 compared to the susceptible RTx430. These defensin genes include Sobic.003G179300, Sobic.008G082300 and three tightly linked duplicate genes (Sobic.003G415200, Sobic.003G415300 and Sobic.003G415800). Interestingly, the up-regulated genes include the sorghum orthologue of the widely known LysM motif receptor kinase (LYK5) (Sobic.004G076100), leucine rich repeat (LRR) receptor-like serine/threonine-protein kinase (Sobic.006G217900) and somatic embryogenesis receptor-like kinase 1 (SERK1) (Sobic.006G104500) all of which were predicted to encode components of the pathogen recognitions and signaling complex. LYK5 is the major chitin receptor in Arabidopsis [14] and hence the sorghum Sobic.004G076100 gene referred here as *SbLYK5*, which encodes a LysM protein is the likely sorghum orthologue with a potential role in recognition of chitin which is a fungal microbe-associated molecular pattern. The sorghum RLK gene (Sobic.006G217900) encodes a putative flagellin receptor (FLS2) with 92.2% amino acid similarity to the maize gene GRMZM2G080041 [15]. FLS2 is a well characterized flagellin receptor in Arabidopsis [16] that plays a critical role in pathogen perception and signaling [15]. The WRKY transcription factor gene (Sobic.004G065900) was up-regulated in RTx2911 and shows similarity to the WRKY71 and WRKY40 genes. The Arabidopsis WRKY40 is a pathogen inducible transcription factor which along with WRKY18 and WRKY60 contributes to defense against pathogens [17]. Jasmonate ZIM domain (JAZ) proteins are transcriptional repressors in jasmonic acid (JA) responses but also play role in regulation of defense-growth balance [18]. The genes (Sobic.001G482700, Sobic.002G214800) up-regulated in RTx2911 that encode JAZ proteins may have similar roles in maintaining defense and growth balance in sorghum. Another gene up-regulated in the resistant genotype (Sobic.003G360900) encodes the isoflavone 2′-hydroxylase which catalyzes steps in phytoalexin biosynthesis pathway and modulates pathogen induced phytoalexin accumulation [19]. Moreover, Sobic.001G373100 gene, up-regulated in RTx2911 encodes ring finger and CHY zinc finger domain-containing protein. Such proteins are involved in diverse biological functions including defense against pathogens [20]. The Sobic.007G030900 gene which is up-regulated in RTx2911 encodes a copine protein that is reported as a possible suppressor of defense responses in Arabidopsis [21]. Copines are conserved calcium-dependent membrane-binding proteins [22]. The Sobic.006G002400 gene which is also up-regulated in RTx2911 encodes amidase family protein. Amidase family proteins are specific indole-3-acetamide amidohydrolase enzymes that catalyze the synthesis of indole-3-acetic acid (IAA) from indole-3-acetamide [23]. IAA is a widely known auxin that regulates plant growth and development. IAA, however, may impact disease resistance negatively [24] which could play a role in balancing immune responses and plant fitness [25]. Another up-regulated gene with a closely related function is Sobic.010G241200 that encodes an IAA-amino acid hydrolase ILR1-Like 6. IAA-amino acid hydrolases cleave IAA-amino acid conjugates releasing free IAA [26]. The sorghum homolog of putative nematode resistance gene Hs1pro-1 [27] (Sobic.003G361100) was also up-regulated in the resistant genotype.

**Table 2:** Defense genes induced at 24 h after inoculation in RTx2911 compared to RTx430

No	Genes	Protein Family	Log_2_ fold change	Test stat	*p*-value	q-value
1	Sobic.001G482700	Jasmonate ZIM	2.26844	3.80456	0.00005	0.000509
2	Sobic.002G214800	Jasmonate ZIM	2.14288	2.59649	0.00045	0.003348
3	Sobic.003G360900	Isoflavone 2′-hydroxylase	2.21360	3.65215	0.00005	0.000509
4	Sobic.001G143000	NPR1/NIM1	1.71135	2.65630	0.00020	0.001696
5	Sobic.001G373100	Ring finger and CHY zinc finger	1.21354	1.62130	0.00990	0.039520
6	Sobic.001G400800	Pathogenesis-related	1.12077	2.22246	0.00035	0.002713
7	Sobic.001G400900	Pathogenesis-related	1.44022	2.22800	0.00085	0.005660
8	Sobic.001G401300	Pathogenesis-related	1.65550	5.60828	0.00005	0.000509
9	Sobic.005G169200	Pathogenesis-related	1.47186	4.52536	0.00005	0.000509
10	Sobic.005G169400	Pathogenesis-related	1.84134	5.27806	0.00005	0.000509
11	Sobic.002G087500	Ricin-type beta-trefoil lectin	3.05161	5.06606	0.00005	0.000509
12	Sobic.003G086200	NPR1 interactor	2.25107	2.13331	0.00625	0.027758
13	Sobic.003G179300	Defensin	3.52422	4.23898	0.00110	0.006982
14	Sobic.003G415200	Defensin	4.32492	5.08045	0.00005	0.000509
15	Sobic.003G415300	Defensin	4.75506	7.44801	0.00005	0.000509
16	Sobic.003G415800	Defensin	2.22990	4.07364	0.00005	0.000509
17	Sobic.008G082300	Defensin	5.38255	5.58025	0.00005	0.000509
18	Sobic.003G233200	NAD dependent epimerase/dehydratase	2.58016	2.08103	0.00010	0.000942
19	Sobic.003G361100	Putative nematode-resistance	1.10019	3.01182	0.00005	0.000509
20	Sobic.004G065900	WRKY DNA binding	1.13824	1.67049	0.00785	0.033027
21	Sobic.004G076100	LYK5, LysM motif receptor kinase	3.53336	2.59092	0.00575	0.026088
22	Sobic.004G317500	(S)-norcoclaurine synthase	2.60706	3.39827	0.00005	0.000509
23	Sobic.005G165700	Plant antimicrobial peptide	2.19914	7.43702	0.00005	0.000509
24	Sobic.006G002400	Amidase family protein	1.11214	3.91534	0.00005	0.000509
25	Sobic.006G083000	Serine/threonine-protein kinase	1.04670	2.54241	0.00005	0.000509
26	Sobic.006G104500	SERK1 (somatic embryogenesis receptor-like kinase 1)	1.15820	2.03747	0.00135	0.008231
27	Sobic.006G217900	FLS2, LRR receptor-like serine/threonine-protein kinase	4.05319	4.17596	0.00005	0.000509
28	Sobic.007G030900	Copine	1.61544	5.03485	0.00005	0.000509
29	Sobic.009G113700	Peroxidase	1.24292	2.05953	0.00080	0.005378
30	Sobic.010G120800	Protein kinase	1.89065	2.33309	0.00140	0.008447
31	Sobic.010G241200	IAA-amino acid hydrolase ILR1-like 6	1.92368	3.26698	0.00005	0.000509

### Gene ontology analyses of molecular processes identify multiple differentially regulated pathways

Significantly enriched GO molecular processes associated with the DEGs in RTx2911 include cofactor binding with 69 genes, oxidoreductase activity with 88 genes, naringenin-chalcone synthase activity with 4 genes, hydrolase activity with 141 genes (Fig. 2, Fig. S2a). Additional DEGs fall with the molecular functions such as DNA binding, carbohydrate binding, protein kinase activity, transcriptional regulation and transmembrane activities. The list of various protein kinase genes up-regulated in RTx2911 are presented in Table S3 which include receptor like kinases, wall associated kinases, and mitogen-activated protein kinases (MAPKs). Similarly, enriched molecular processes associated with the down-regulated genes include chlorophyll binding with 9 genes, cofactor binding with 57 genes, and oxidoreductase activity with 76 genes (Fig. 2, Fig. S2b). Hydrolase activity, carbohydrate binding, and catalytic activity acting on protein constitute the top three categories of processes represented by the upregulated genes in the resistant genotype (Fig. S2a). Similarly, anion binding, small molecule binding, and oxidoreductase were processes that were represented by a larger proportion of down-regulated genes (Fig. S2b).

### KEGG enrichment analysis of metabolic pathways for DEGs

**Fig. 3:**
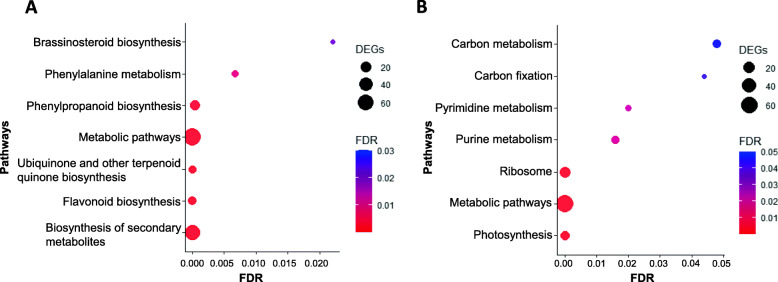
KEGG pathways for genes differentially expressed between genotypes. **a** Enriched KEGG pathways for genes with higher induced expression in RTx2911 at 24 hpi compared to RTx430. **b** Enriched KEGG pathways for genes down-regulated in RTx2911 at 24 hpi compared to RTx430

KEGG enrichment analysis using genes up-regulated in RTx2911 at 24 hpi identified significantly enriched metabolic pathways associated with grain mold resistance. These include biosynthesis of flavonoid (11 genes), other secondary metabolites (53 genes), ubiquitin and other terpenoid-quinone, phenylpropanoid, phenylalanine and brassinosteriods (Fig. 3a). The up-regulated flavonoid biosynthesis genes include 4 chalcone synthase (Sobic.005G136200, Sobic.005G136300, Sobic.005G137000, and Sobic.005G137300), chalcone-flavonone isomerase (Sobic.001G035600), cytochrome P450 (Sobic.002G126600), flavonoid 3′-hydroxylase (Sobic.004G200900), glucosyl/glucuronosyl transferase (Sobic.007G027301), shikimate O-hydroxycinnamoyl transferase (Sobic.006G136800), bifunctional dihydroflavonol 4-reductase/flavanone 4-reductase (DFR, Sobic.004G050200) and cinnamate 4-hydroxylase (Sobic.004G141200) genes. On the other hand, down-regulated genes were in photosynthesis (11 genes), ribosome, purine, pyrimidine, and carbon metabolism functions (Fig. 3b).

### Pathogen induced differential expression of genes in developing sorghum grain

**Fig. 4:**
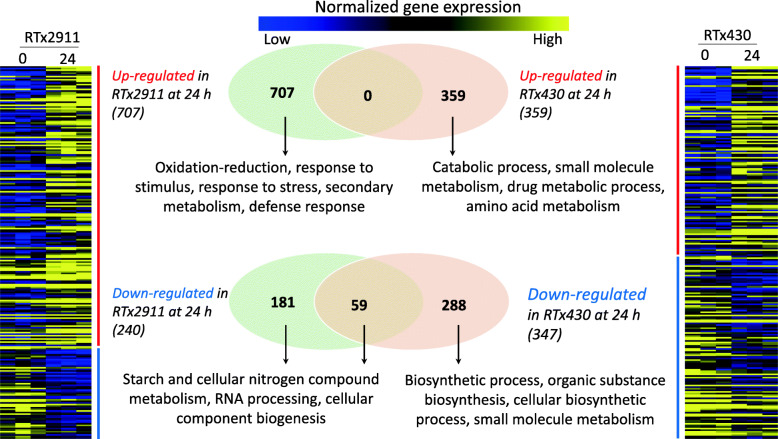
Differentially expressed genes regulated by pathogen inoculation in grain mold resistant and susceptible genotypes and significantly enriched GO terms. GO terms displayed indicate  those with highest significance

In order to decipher DEGs induced in response to fungal inoculation, transcriptome comparisons were made between samples prior to and after inoculation for each genotype. A number of genes were differentially expressed in response to fungal inoculation (Fig. 4). Consequently, 947 DEGs with altered expression in response to inoculation were identified in RTx2911 with 707 up-regulated and 240 down-regulated at 24 hpi compared to 0 hpi (Fig. 4, Table S4). Similarly, 706 genes were differentially expressed between the two time points in RTx430 with 359 genes up-regulated and 347 down-regulated at 24 hpi compared to 0 hpi (Fig. 4, Table S5). Among these, 59 genes were down-regulated at 24 hpi in both RTx2911 and RTx430 compared to basal expression at 0 hpi.

GO enrichment analysis of genes differentially expressed between the time points for each genotype revealed significantly enriched biological, chemical, cellular and KEGG pathways. This analysis which compares differentially expressed genes between 0 (before infection) and 24 h (after infection) indicates genes that are particularly induced upon infection in each of the genotypes. The complete list of significantly enriched biological processes and number of genes under each process are presented as Fig. S3a-d).

Genes up-regulated after infection in RTx2911 were assigned mainly to defense associated biological processes such as response to stimulus (101 genes), stress (62 genes), defense (24 genes), chitin response (3 genes), biotic stimulus (19 genes), oxidation-reduction, carbohydrate metabolism, secondary metabolism, flavonoid biosynthesis and others (Fig. 4, Fig. S3a). Genes induced upon infection in RTx2911 that were also differentially expressed between the two genotypes include a JAZ repressor (Sobic.001G482700), isoflavone 2′-hydroxylase (Sobic.003G360900), ring finger and CHY zinc finger (Sobic.001G373100), nematode-resistance (Sobic.003G361100), WRKY DNA binding (Sobic.004G065900), and *SbLYK5* (Sobic.004G076100) genes (Table 3).

**Table 3:** Defense genes induced upon infection in RTx2911 at 24 h post inoulation

No	Genes	Protein Family	Log_2_ fold change	Test stat	p-value	q-value
1	Sobic.001G482700	Jasmonate ZIM	1.02671	2.02240	0.00055	0.008999
2	Sobic.003G360900	Isoflavone 2′-hydroxylase	1.23157	2.26308	0.00075	0.011313
3	Sobic.001G077400	Allene oxide synthase	1.49778	2.72515	0.00010	0.002305
4	Sobic.001G156100	Ornithine aminotransferase	1.29051	4.01677	0.00005	0.001305
5	Sobic.001G165600	Defensin	1.23670	2.56264	0.00005	0.001305
6	Sobic.005G153600	Defensin	1.14584	2.57529	0.00005	0.001305
7	Sobic.007G075250	Defensin	1.68390	4.82551	0.00005	0.001305
8	Sobic.007G075301	Defensin	1.93758	5.78103	0.00005	0.001305
9	Sobic.001G373100	Ring finger and CHY zinc finger	1.71346	2.06206	0.00235	0.025444
10	Sobic.001G400700	Pathogenesis-related	1.26150	1.74839	0.00370	0.034773
11	Sobic.001G400800	Pathogenesis-related	1.42753	2.49106	0.00005	0.001305
12	Sobic.001G400900	Pathogenesis-related	2.86101	3.23454	0.00005	0.001305
13	Sobic.001G401100	Pathogenesis-related	3.26565	2.91294	0.00125	0.016513
14	Sobic.001G401200	Pathogenesis-related	4.12443	5.39278	0.00005	0.001305
15	Sobic.001G401300	Pathogenesis-related	2.19583	5.97457	0.00005	0.001305
16	Sobic.005G169200	Pathogenesis-related	4.41356	7.95931	0.00005	0.001305
17	Sobic.005G169300	Pathogenesis-related	1.70302	4.90227	0.00005	0.001305
18	Sobic.005G169400	Pathogenesis-related	2.91304	6.29651	0.00005	0.001305
19	Sobic.003G361100	Putative nematode-resistance	1.52266	3.49796	0.00005	0.001305
20	Sobic.003G379700	NAC23 (GRAB1 like protein)	1.06726	3.28441	0.00005	0.001305
21	Sobic.004G065900	WRKY DNA binding	1.47453	1.88306	0.00495	0.042569
22	Sobic.004G076100	LYK5, LysM motif receptor kinase	1.67926	1.91315	0.00325	0.031605
23	Sobic.006G005600	Heat shock protein	2.98005	5.59362	0.00005	0.001305
24	Sobic.007G194800	Triacylglycerol lipase 2	1.24681	3.73981	0.00005	0.001305

**Fig. 5:**
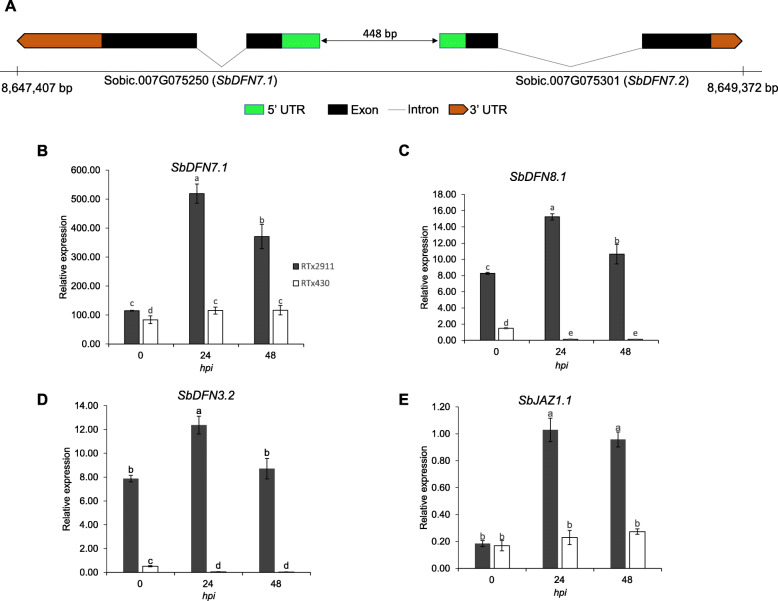
Genomic organization of defensin genes and validation of expression of selected genes through quantitative qRT-PCR. **a** Schematic representation of the genomic organization of defensin genes *SbDFN7.1* and *SbDFN7.2* on chromosome 7 in sorghum. Quantitative reverse transcription-PCR (qRT-PCR) analysis of selected *Defensin (gamma-thionin)* (**b-c**) and Jasmonate ZIM domain (JAZ) genes (**d**). hpi, hours post inoculation

Moreover, cytochrome P450 (Sobic.001G077400), ornithine aminotransferase (Sobic.001G156100), defensins (Sobic.001G165600, Sobic.005G153600, Sobic.007G075250, Sobic.007G075301), pathogenesis-related (9 genes), the NAC protein geminivirus rep a-binding 1 (GRAB1) (Sobic.003G379700), heat shock (Sobic.006G005600) and triacylglycerol (TAG) lipase (Sobic.007G194800) genes were induced upon infection in the resistant genotype (Table 3). The Sobic.001G077400 gene encodes an allene oxide synthase (AOS), which is a cytochrome P450 protein. The sorghum putative AOS shares high similarity (98%) to the maize hydroperoxide dehydratase. AOS shows hydroperoxide dehydratase activity which catalyzes the first step in the biosynthesis of jasmonic acid, a major regulator of plant defense to necrotrophic fungal pathogens [28]. High level of such enzymes accumulate in pericarps and seed coats [29] which suggests their important roles in defense against grain pathogens. The Sobic.001G156100 gene encodes a highly conserved enzyme, ornithine aminotransferase which contributes to both R-gene mediated and non-host resistance through proline metabolic pathway [30, 31]. Four defensin genes (Sobic.001G165600, Sobic.005G153600, Sobic.007G075250, Sobic.007G075301) were induced upon infection in the resistant genotype. Two of these genes (Sobic.007G075250, refereed here as *SbDFN7.1*; Sobic.007G075301, refereed here as *SbDFN7.2*) are tightly linked on sorghum chromosome 7, and transcribed in opposite orientation with a likely common promotor (Fig. 5a). *SbDFN7.1* and *SbDFN7.2* are similar to maize *ZmDEF1* (GRMZM2G368890) and *ZmDEF2* (GRMZM2G368861) in both genomic organization and sequence similarity. The intergenic region of the maize defensin genes *ZmDEF1* and *ZmDEF2* is considered as an embryo-specific asymmetric bidirectional promoter [32]. These defensin genes are specifically and highly expressed in seeds. Plant defensins are pathogen inducible [33] antimicrobial peptides [13]. The Sobic.003G379700 which encodes a NAC transcription factor was induced upon infection in RTx2911 and shares high similarity (97%) to the maize GRAB1-like protein. NAC transcription factors play role in regulation of biotic and abiotic stress responses [34] while the GRAB1 proteins which are members of the NAC domain family are known for their interaction with a geminivirus protein [35]. The other induced gene in RTx2911, Sobic.006G005600, encodes a heat shock protein (HSP90). HSP90 is the most abundant cytosolic heat shock protein family [36] and plays important roles in immune responses [37, 38]. The Sobic.007G194800 gene induced in RTx2911 encodes an important protein TAG lipase, which is similar to the Arabidopsis *phytoalexin deficient 4* (PAD4) [39]. Plants with *pad4* mutations display defects in multiple defense responses with reduced camalexin synthesis, PR-1 gene expression and SA levels [40]. This is interesting because sorghum produces the phytoalexin 3-Deoxyanthocyanidins which accumulate in response to fungal infection [41]. 3-Deoxyanthocyanidins are synthesized through the flavonoid biosynthesis pathway.

Genes up-regulated in RTx430 upon infection were assigned to catabolic process, small molecule metabolism, drug metabolic process, cellular homeostasis, response to biotic stimulus (9 genes) and defense (10 genes) (Fig. 4, Fig. S3b). The 10 defense genes up-regulated in RTx430 include 2 genes that were specifically induced in RTx430, which are an NBS-LRR resistance gene (Sobic.005G092600) and l-type lectin-domain containing receptor kinase (Sobic.004G118800) and 8 were similar to that of RTx2911. The 8 genes commonly up-regulated in both RTx2911 and RTx430 are ornithine aminotransferase (Sobic.001G156100), 5 PR genes (Sobic.001G400800, Sobic.001G401100, Sobic.001G401200, Sobic.001G401300, Sobic.005G169400), HSP90 (Sobic.006G005600) and TAG lipase (Sobic.007G194800).

Genes down-regulated upon infection in RTx2911 were assigned to starch metabolism, cellular nitrogen compound metabolism, RNA processing, DNA metabolic process and others (Fig. 4, Fig. S3c). On the other hand, genes down-regulated in RTx430 upon infection were assigned to biosynthetic process, organic substance biosynthesis, cellular biosynthesis process, small molecule metabolism, oxidation-reduction process, secondary metabolism biosynthesis and others (Fig. 4, Fig. S3d).

### Increased expression of genes encoding seed storage proteins in grain mold resistant sorghum

A major variation between RTx430 and RTx2911 was observed in expression of genes encoding seed storage proteins. Sobic.005G184500 annotated as zein seed storage protein was the most variable between the two genotypes in terms of expression both prior to and after inoculation (Tables S1 & S2). This gene showed higher expression in RTx2911 with a Log2 fold change of 9.7 in non-inoculated grain and 10.7 at 24 h after inoculation. Sobic.008G144201 was another gene with higher basal and pathogen induced expression in RTx2911 that also encodes a zein seed storage protein. Both Sobic.005G184500 and Sobic.008G144201 were highly expressed in developing grain of the resistant genotype RTx2911. Zein and kafirins are major seed storage proteins in maize and sorghum, respectively, which are associated with kernel texture [42–44]. Recently, a major kafirin locus was discovered as key determinates of grain mold resistance in sorghum [45].

### Validation of differential expression of selected defense genes using qRT-PCR

To validate the sequence data and also determine expression pattern of some genes beyond the two time points used for RNA-seq, expression of selected genes encoding defensins and a JAZ protein genes were studied using qRT-PCR. Broadly, the resistant genotype showed a significantly higher level of expression than the susceptible genotype (Fig. 5). The expression of the sorghum defensin genes *SbDFN7.1* (Sobic.007G075250), *SbDFN8.1* (Sobic.008G082300), *SbDFN3.2* (Sobic.003G415300) (Fig. 5b-d) and the *SbJAZ1.1* gene (Sobic.001G482700) were consistent with those observed in RNA-seq. All the three genes that encode the sorghum defensins were highly induced at 24 hpi in the resistant genotype RTx2911. At 48 hpi, the expression of these genes varied slightly with *SbDFN7.1* (Fig. 5b) and *SbDFN8.1* (Fig. 5c) but remained higher than that of 0 hpi but slightly lower than 24 hpi whereas the expression of *DFN3.2* at 48 hpi leveled to the 0 hpi (Fig. 5d). The expression of the *SbJAZ1.1* gene increased significantly at 24 hpi and remained high at 48 hpi (Fig. 5e). The expression of these genes in the susceptible RTx430 was lower at 0 hpi which either remained low (*SbDFN7.1* and *SbJAZ1.1*) or was reduced to a very low expression level at 24 and 48 hpi (*SbDFN8.1* and *SbDFN3.2*).

## Discussion

This study focused on transcriptome changes in the developing grain in response to simultaneous infection by grain molding fungal species. Defense responses in grain tissues to single or a mixture of multiple pathogenic species have not been studied previously. Responses to a mixture of fungi rather than a single species mirrors sorghum grain mold disease in the field under natural infestations. RNA was extracted from the developing grain before and after the genotypes were challenged by a mixture of spore suspension of five *Fusarium* and an *Alternaria* species. The sampling time points 0, 24 and 48 h after inoculation were based on our previous study on grain [46] and leaf [47] that defense related genes are induced within 24 to 48 h after inoculation. RNA samples from two of the time points (0 and 24 h) were used for sequencing as preliminary studies indicated that defense genes were already induced at 24 h after infection while the 48 h samples were used for validation studies. Global changes in gene expression, molecular and cellular functions, and metabolic pathways that are reprogrammed early during infection of the developing grain were delineated, which together are likely to explain variations in plant responses to the disease. Comparative transcriptome and subsequent gene ontology enrichment analysis in resistant and susceptible sorghum genotypes revealed differentially expressed genes that are associated with major plant defense pathways, seed proteins and antimicrobial protein genes that were preferentially expressed in the resistant genotype. Genes that showed higher basal and induced gene expression in the resistant genotype relative to the susceptible genotype are implicated in key plant defense pathways. Antimicrobial peptides including plant defensins and genes that encode proteins that preferentially accumulate in the seed but are also induced in response to infection were identified. This is consistent with the role of seed proteins and other compounds that regulate the physical and chemical properties of kernels, and thus provide resistance to grain mold. Interestingly, we also observed differential expression of genes encoding proteins that function in pathogen recognition, signal transduction, and other defense responses sharing similarity to immune mechanisms in leaf tissues in many plant pathogen interactions.

The major defense related genes induced in the resistant genotype RTx2911 in response to infection include PR proteins, antimicrobial peptides including defensins, receptor like kinases, regulators of systemic acquired resistance (SAR) and biosynthesis of phytoalexins as well as genes known to be involved in flavonoid biosynthesis. Analyses of enriched molecular processes identified components of pathogen recognition and response signaling such as receptor like protein kinases, wall associated kinases and mitogen-activated protein kinases (MAPKs). Thus, resistance to grain mold in developing sorghum grain involves active defense processes that involve recognition of pathogen or damage associated molecular patterns by plant receptors followed by activation of signal transduction pathways that trigger multiple immune responses consistent with the quantitative nature of grain mold resistance. Such active defense response pathways likely culminate in synthesis of antimicrobial molecules, changes in seed protein profile, and enhancement of seed physical and biochemical defenses which may be superimposed on passive defense mechanisms.

PAMP triggered immunity (PTI) to pathogens is a form of quantitative resistance that is initiated by perception of evolutionarily conserved pathogen derived molecules, such as chitin fragments, by surface localized pattern recognition receptors (PRRs) [48]. The induced expression of the sorghum LysM motif receptor kinase (*SbLYK5*) in response to infection in the resistant genotype RTx2911 is consistent with the activation of PTI. The Arabidopsis *AtLYK5* is the receptor for chitin and is also chitin inducible [14] suggesting the sorghum orthologue identified in our study may have similar functions. Sorghum 3-deoxyanthocynidin, phytoalexins synthesized through the flavonoid pathway, and known to accumulate in response to pathogen infection may be activated by perception of fungal derived chitin fragments by *SbLYK5*. The fact that several flavonoid biosynthesis genes were induced upon infection in our study, and the co-expression of PRRs supports that the phytoalexin biosynthesis branch of the flavonoid biosynthesis pathway may be correlated with chitin perception and response signaling in the developing grain. Perception of pathogen derived elicitor by membrane localized PRRs, and their subsequent response signaling by their downstream components such as receptor like cytoplasmic kinases (RLCK) and MAPKs are known to contribute to activation of defense responses [49–51]. The enhanced expression of sorghum genes encoding putative PRRs, RLCKs and MAPKs in the resistant genotype suggest the role of PTI mechanisms in restricting the severity of grain mold in the developing grain. The data also suggest that in the developing grain that is at the physiologically active stage, the induced immune mechanism may contribute significantly, which may decline after the grain is physiologically mature when physical or passive mechanisms are likely to supersede.

Different pathogenies-related (PR) genes with higher basal and induced expression in the resistant than the susceptible genotype suggest their critical roles in resistance against grain mold in sorghum. PR-related proteins are conserved protein families involved in plant immunity [52, 53] some of which are involved in both biotic and abiotic stress responses [54]. The PR genes identified in this study occur as clusters of duplicates in two loci in sorghum which are located at 68.6 and 64.8 Mbp on chromosome 1 and 5, respectively. Those on Chromosome 1 encode proteins similar to the Bet v I family of PR-10 and those on Chromosome 5 encode chitinase-related proteins. PR-10 proteins have ribonuclease activities [55, 56]. Chitinases accumulate in response to stress or pathogen attack [57]. Some PR genes identified in the current study were induced upon infection in both the resistant and the susceptible genotypes but some were only induced in the resistant genotype.

Our data suggest that defensins which are small (~ 5 kDa) basic, cysteine-rich antimicrobial peptides [13, 58] are among the major elements of the sorghum defense system that are induced in response to grain mold fungi that are typically necrotrophic pathogens. Plant defensins are classified as PR-12 family proteins [59, 60] and are components of the plant immune response especially to necrotrophic fungi [58, 61] with high fungi toxic activities [62] and the majority of defensins reported accumulate in the seed [12]. Several genes encoding these peptides were highly induced upon infection in the resistant genotype RTx2911 but their expression was severely attenuated in the susceptible RTx430. Defensin expression is dependent on functional ethylene and jasmonic acid response pathways [33]. A cytochrome P450 gene encoding allene oxide synthase (AOS) which is involved in the biosynthesis of JA [28] was induced upon infection in the resistant genotype. JA is associated with defense against necrotrophic fungi [63] and may play critical regulatory roles in the activation of defenses against grain mold in sorghum including the expression of defensins that require JA perception and signaling. Sorghum defensins have not been studied as they are mostly grain specific and pathogen inducible whereas most previous studies focus on foliar tissues. It is notable that defensins were not prominently described in recent RNA-seq experiments conducted in leaf tissues of sorghum consistent with their grain specific expression [47, 64].

Among major and widespread plant defense responses to pathogens are the pathogen induced accumulation of phytoalexins, which are low molecular weight antimicrobial compounds [65, 66]. Sorghum produces the 3-Deoxyanthocyanidin phytoalexins, apigeninidin and luteolinidin [67] through the flavonoid biosynthesis pathway. Indeed our study indicated that several flavonoid biosynthesis pathway genes were differentially expressed between the resistant RTx2911 and susceptible RTx430, a subset of which were also induced upon infection in the resistant genotype. The TAG lipase protein gene which is similar to Arabidopsis PAD4, was induced upon pathogen inoculation in both resistant and susceptible genotypes. Looking at a previous study conducted on the biosynthesis pathway of camalexin and the nature of the enzymes involved, it seems that the camalexin biosynthesis pathway has some level of similarity to that of the sorghum cyanogenic glycoside dhurrin [68]. Metabolite profiling of developing grain also indicated that dhurrin accumulates in early stage of grain development reaching maximum amounts at 25 days after flowering but the grains were acyanogenic as demonstrated by lack of hydrogen cyanide and absence of transcripts encoding dhurrinases [69]. However, there is no evidence suggesting antimicrobial effect of dhurrin or hydrogen cyanide, which are generated during dhurrin biosynthesis.

GO enrichment analysis suggested that genes associated with photosynthesis were negatively regulated in the resistant genotype suggesting suppression of photosynthesis during enhanced defense responses. Therefore, disease resistant genotypes with good agronomic performance may harbor mechanisms that maintain the balance between defense and growth. Up-regulation of some genes that repress defense responses in the absence of pathogens is part of such mechanism. The plant hormone JA regulates inducible defenses, and plays a crucial role in growth-defense tradeoffs by regulating carbon assimilation and partitioning [70]. Interestingly, the resistant genotype shows induced expression of transcriptional repressors of JA and/ or defense responses such as JAZ proteins. Accumulation of JA in response to infection or other environmental cues promotes degradation of JAZ proteins that relieves repression on various transcription factors [18]. JAZ proteins suppress accumulation of anthocyanin by interacting with WD-Repeat/bHLH/MYB complexes while JA-induced degradation of JAZ proteins eliminates the interaction [71].

**Fig. 6:**
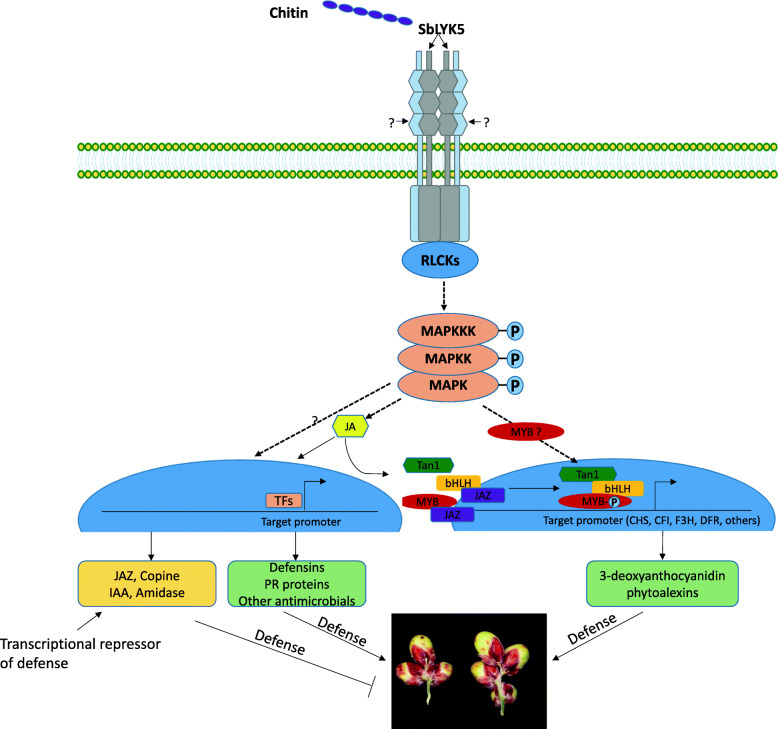
Proposed model for pathogen inducible defense system in sorghum grain and major components. The model depicts how a putative sorghum chitin receptor (SbLYK5) and an unknown coreceptor could be activated by perception of chitin that triggers a pathogen response signaling involving RLCKs, MAPKs, and various potential transcriptional regulators. TFs, transcription factors; CHS, chalcone synthase; CFI, Chalcone flavanone isomerase; F3H, Flavonoid 3′-hydroxylase; DFR, dihydroflavonol 4-reductase

Pathogen inducible defense against major crop diseases is a vital component of resistance which may have less effect on resources that would rather be allocated to growth in the absence of pathogens. Although parts of this system is known in sorghum and other crop plants, regulation of pathogen induced defense mechanism is poorly understood. For instance, although the roles of sorghum 3-deoxyanthocyanidin phytoalexins in defense are known and that they are pathogen inducible but the upstream regulatory mechanisms that link pathogen perception to downstream target genes is unknown. In this regard, genetic evidence shows that the sorghum *Y1* and the *Tan1* genes are associated with resistance to grain mold [45, 46] and these genes regulate the biosynthesis of 3-deoxyanthocyanidin phytoalexins but the molecular link between pathogen perception and biosynthesis of the phytoalexins are not known. Based on evidences from this study and previous reports, we provide a conceptual model of pathogen inducible defense system in sorghum (Fig. 6). As described in the preceding sections, we identified the *SbLYK5* gene that encodes a receptor like kinase that may function as the sorghum chitin receptor. *SbLYK5* and other RLKs likely serve as receptor complexes, and their downstream components such as RLCKs and MAPK are recruited in pathogen response signaling leading to gene expression and accumulation of defense active secondary metabolites. This is consistent with data from rice and Arabidopsis where MAPK cascades and their downstream transcription factors regulate phytoalexin biosynthesis [72]. Interestingly, a recent report suggests an R2R3 MYB transcription factor is phosphorylated by MPK4 which is required for light induced anthocyanin accumulation in Arabidopsis [73]. We therefore, speculate that the sorghum R2R3 MYB proteins encoded by *Y1* may be phosphorylated by an unidentified MPK or RLCKs in sorghum and play role in signaling of pathogen responses and accumulation of secondary metabolites. The figure summarizes our working model of how the biosynthesis of 3-deoxyanthocynidin phytoalexins, defensins, PR proteins, and other antimicrobial peptides as well as defense suppressing proteins may be regulated through pathogen inducible defense system in sorghum grain.

## Conclusion

Grain is a distinct tissue from the widely studied leaf tissue, contains rich carbon source that makes it prone to infection. Despite the importance of grain as the final and most valuable product of the crop production effort, genetic resistance and the status of defense responses in the grain have been poorly studied. Transcriptome profiling in the developing grain of sorghum genotypes revealed both conserved and unique defense mechanisms that may underlie differences in resistance to the disease. Differential expression of regulators of quantitative resistance were found to correlate with resistance in early stages of sorghum grain. In addition, JA response and biosynthesis pathways showed differential expression correlating with resistance extending the role of these plant hormone to grain tissue and complex diseases. These observations suggest that many responses in the grain are regulated by similar mechanisms that are active in leaf tissue despite the distinct nature of the leaf and grain tissues. By contrast, genes encoding pathogenesis-related proteins, defensins, phytoalexins and zein seed storage proteins, that are uniquely regulated in grain, and pathogen infection showed higher basal and induced expression in the resistant genotype. Interestingly, previously undescribed sorghum defensin genes that are induced upon infection or were constitutively expressed at a higher level in the resistant genotype were also identified. Further, we provide new insights into molecular, cellular, and biochemical processes underlying response to a complex disease involving a consortium of necrotrophic fungi with aggressive pathogenesis strategies, as well as a host resistance with complex genetic architecture. Potential regulators of sorghum pathogen recognition at the very early stages of attempted infection, and downstream genetic components of defense that may have antibiotic activities, or molecules that reinforce the grain structure to make it impermeable to pathogen ingress were identified. Together, the newly identified components may contribute to grain mold resistance and provide new insights into an understudied pathosystem, and will serve as targets for genetic studies and to identify resistance germplasm.

## Materials and methods

### Plant materials

RTx2911 which is resistance to grain mold [74] and RTx430, highly susceptible to the disease [75]. The resistance and susceptibility reaction of the two genotypes to a number of grain mold causing fungal species has been confirmed in a serious of greenhouse (humidity chamber) based experiments that we have conducted recently [46].

#### Inoculation of the developing sorghum grain with grain mold fungi

A mixture of spore suspension from five *Fusarium* (*F. proliferatum, F. graminearum, F. thapsinum*, *F. verticillioides* and *F. oxysporum*) and one *Alternaria* species were spray inoculated on to panicles of both RTx2911 and RTx430 at 20 days after anthesis. Inoculation and disease establishment were conducted in a humidity chamber equipped with a humidifier that has adjustable humidistat to retain humidity at required level (85–90%). Details of isolation, fungal species identification through sequencing of the ribosomal internal transcribed spacer (ITS) region of fungal DNA, multiplication and inoculation of the fungal species used in this study were described previously [46].

#### Total RNA extraction

RNA was extracted from the developing grain before and after the two genotypes were challenged by a mixture of spore suspension of equal proportions of the five *Fusarium* and an *Alternaria* species. The sampling time points were 0, 24 and 48 h after inoculation. Total RNA was extracted as described [76] with minor modifications [46].

#### Library construction and sequencing

RNA samples were cleaned and concentrated using RNA Clean and Concentrator™ − 25 Kit (ZYMO RESEARCH). The quality of the RNA was evaluated using NanoDrop and Agilent Bioanalyser (RNA Plant Nano, DNA High Sensitivity and RNA Eukaryote Pico Chips). RiboZero libraries were constructed from RNA samples at 0 and 24 h time after inoculation. Then, the libraries were sequenced on an Illumina HiSeq 2500 ultra-high-throughput sequencing system with 150 bp paired-end reads. Since preliminary gene expression analysis of previously described pathogen inducible genes through real time quantitative RT-PCR (qRT-PCR) revealed induction of genes within 24 h after inoculation, the 48 h samples were not included in RNA-seq but used to study expression of individual genes through qRT-PCR.

#### Sequence data filtering and QC

Raw reads were filtered by clipping adaptors, removing low quality reads and duplicated sequences. Sequence quality was assessed by FastQC both before and after the reads were filtered for adaptors, low quality reads and duplicated sequences. Moreover, sequence GC% assessment, rRNA and phiX database matches and organism inference was conducted.

#### Differential gene expression analysis with HISAT and cufflinks

Following data filtering and QC, the resulting high quality clean reads were used to perform differential gene and transcript expression analysis as described [77] with some modifications. The modifications include use of HISAT [78] to align the reads to the sorghum (BTx623) (PhytozomeV12: *Sorghum bicolor v3.1.1*.) and the RTx430 reference genomes instead of TopHat and all job scripts were written in python which provided more efficiency. With large number of samples, instead of writing multiple scripts which can be time consuming, a single python script was applied to automatically generate and execute multiple job scripts for all input samples. Details of the workflow and python scripts used in the current study is provided as supplemental method that can be adopted for similar analysis. List of differentially expressed genes were written out from the Cuffdiff analysis output and genes with a log fold change (LogFC) above one (2-fold change) were used for functional classification and metabolic pathway analysis.

### Hierarchical clustering

To assess variability among samples, hierarchical clustering analysis was performed based on Euclidean distances using WebMeV (Multiple Experiment Viewer) (http://mev.tm4.org). HeatMaps of the samples based on normalized expression values also generated using WebMeV.

#### Functional annotation and metabolic pathway analysis

Using the graphical enrichment tool ShinyGo v0.61 [79], the lists of differentially expressed genes from Cuffdiff analysis were annotated for their underlying biological process, molecular function and cellular component ontology. Metabolic pathway analysis was performed using the ShinyGo tool that also produces KEGG pathway.

#### Validation of gene expression through real time quantitative PCR

Expression of selected genes were validated via qRT-PCR as described [80]. cDNA was synthesized from 2 μg total RNA using the AMV reverse transcriptase (NEB). Quantitative PCR was performed on LightCycler® 96 system (Roche) using a SYBR Green Supermix (Bio-Rad). Sorghum *Actin* gene was used as a constitutive endogenous control. A minimum of three technical and three biological replicates were used for qRT-PCR analysis for each sample. Expression levels were calculated by the comparative C_T_ method [81]. Primers used for qRT-PCR analysis are listed in Table S6.

## Supplementary Information

**Additional file 1 Fig. S1**. Gene Ontology enrichment analysis of DEGs between RTx2911 and RTx430 at 24 hpi. Enriched GO biological process for up (**a**) and down (**b**) regulated genes at 24 hpi in RTx2911 compared to RTx430.

**Additional file 2 Fig. S2**. Gene Ontology enrichment analysis of DEGs between RTx2911 and RTx430 at 24 hpi. **a** Enriched GO molecular process of up-regulated genes at 24 hpi in RTx2911 compared to RTx430. **b** Enriched GO molecular process of down-regulated genes at 24 hpi in RTx2911 compared to RTx430.

**Additional file 3 Fig. S3**. Enriched GO biological processes between 0 and 24 hpi for RTx2911 and RTx430. **a** Up-regulated genes at 24 hpi in RTx2911 compared to 0 hpi. **b** Up-regulated genes at 24 hpi in RTx430 compared to 0 hpi. **c** Down-regulated genes at 24 hpi in RTx2911 compared to 0 hpi. **d** Down-regulated genes at 24 hpi in RTx2911 compared to 0 hpi.

**Additional file 4 Table S1**. Genes differentially expressed between genotypes at 0 hpi

**Additional file 5 Table S2**. Genes differentially expressed between genotypes at 24 hpi

**Additional file 6 Table S3**. Enriched GO molecular process for genes differentially expressed between genotypes: list and description of protein kinase genes up regulated in RTx2911 at 24 hpi

**Additional file 7 Table S4**. Genes differentially expressed between 0 and 24 hpi in RTx2911

**Additional file 8 Table S5**. Genes differentially expressed between 0 and 24 hpi in RTx430

**Additional file 9 Table S6**. List of primers used for qRT-PCR

**Additional file 10.** Details of the workflow and python scripts used to conduct differential gene expression analysis

## Data Availability

The RNA-seq data have been submitted to NCBI and can be accessed via the following link: https://www.ncbi.nlm.nih.gov/sra/PRJNA692482

## Abbreviations

AOS: allene oxide synthase
DEGs: differentially expressed genes
GO: gene ontology
GRAB1: geminivirus rep a-binding 1
hpi: hours post inoculation
KEGG: kyoto encyclopedia of genes and genomes
LogFC: log fold change
MAPKs: mitogen-activated protein kinases
PR: pathogenesis-related
PRRs: pattern recognition receptors
PTI: PAMP triggered immunity
qRT-PCR: quantitative real-time PCR
RLCK: receptor like cytoplasmic kinases
TAG: triacylglycerol
WebMeV: multiple experiment viewer
