# Supplementary material for: Transcriptome analysis of early stages of sorghum grain mold disease reveals defense regulators and metabolic pathways associated with resistance
Source: BMC Genomics. 2021 Apr 22;22:295. doi: 10.1186/s12864-021-07609-y (PMC8063297; doi:10.1186/s12864-021-07609-y)
Supplement: Supplementary file 1 — Additional file 1 Fig. S1. Gene Ontology enrichment analysis of DEGs between RTx2911 and RTx430 at 24 hpi. Enriched GO biological process for up (a) and down (b) regulated genes at 24 hpi in RTx2911 compared to RTx430. [file 12864_2021_7609_MOESM1_ESM.pptx]

## Slide 1
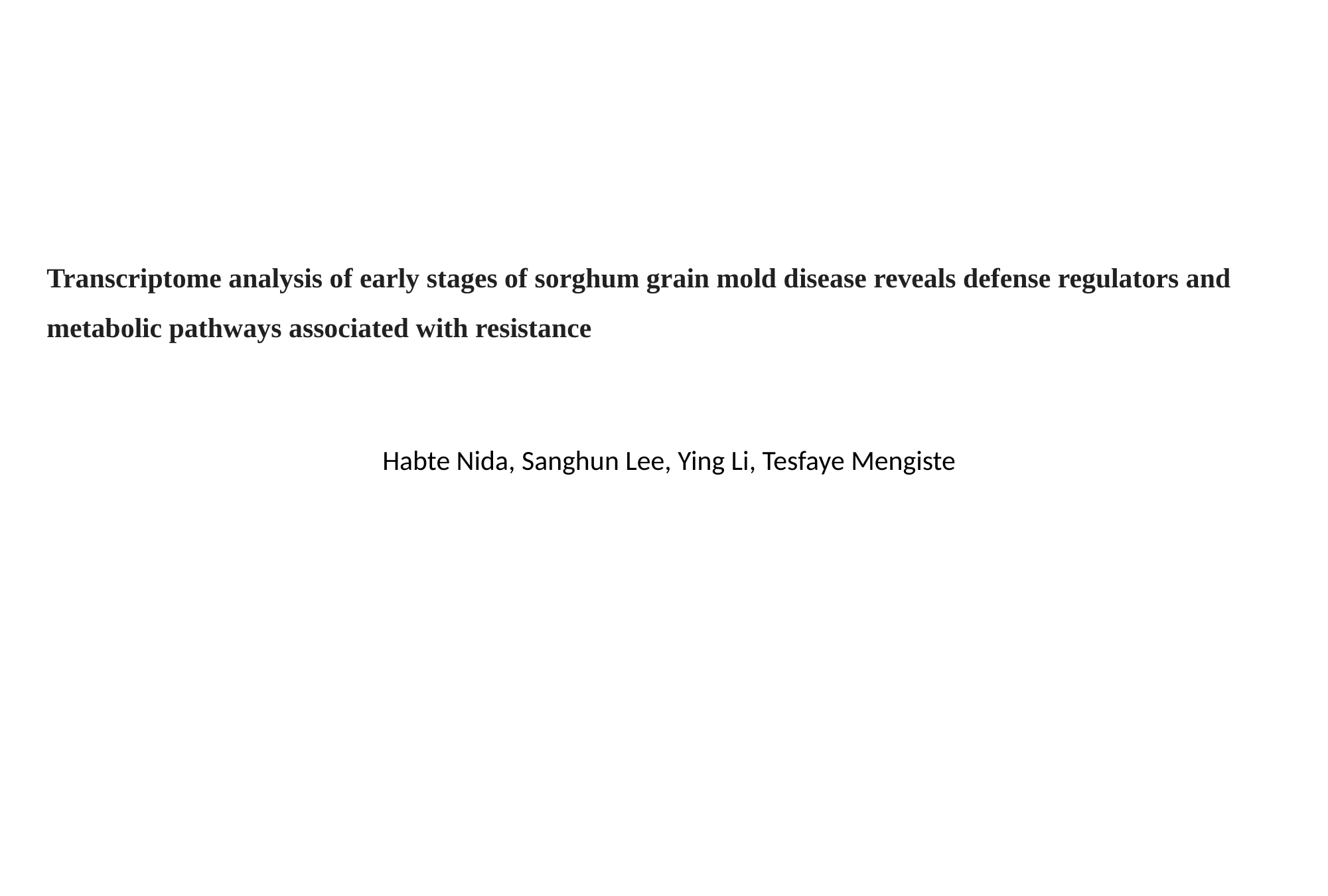

Transcriptome analysis of early stages of sorghum grain mold disease reveals defense regulators and metabolic pathways associated with resistance
Habte Nida, Sanghun Lee, Ying Li, Tesfaye Mengiste

## Slide 2
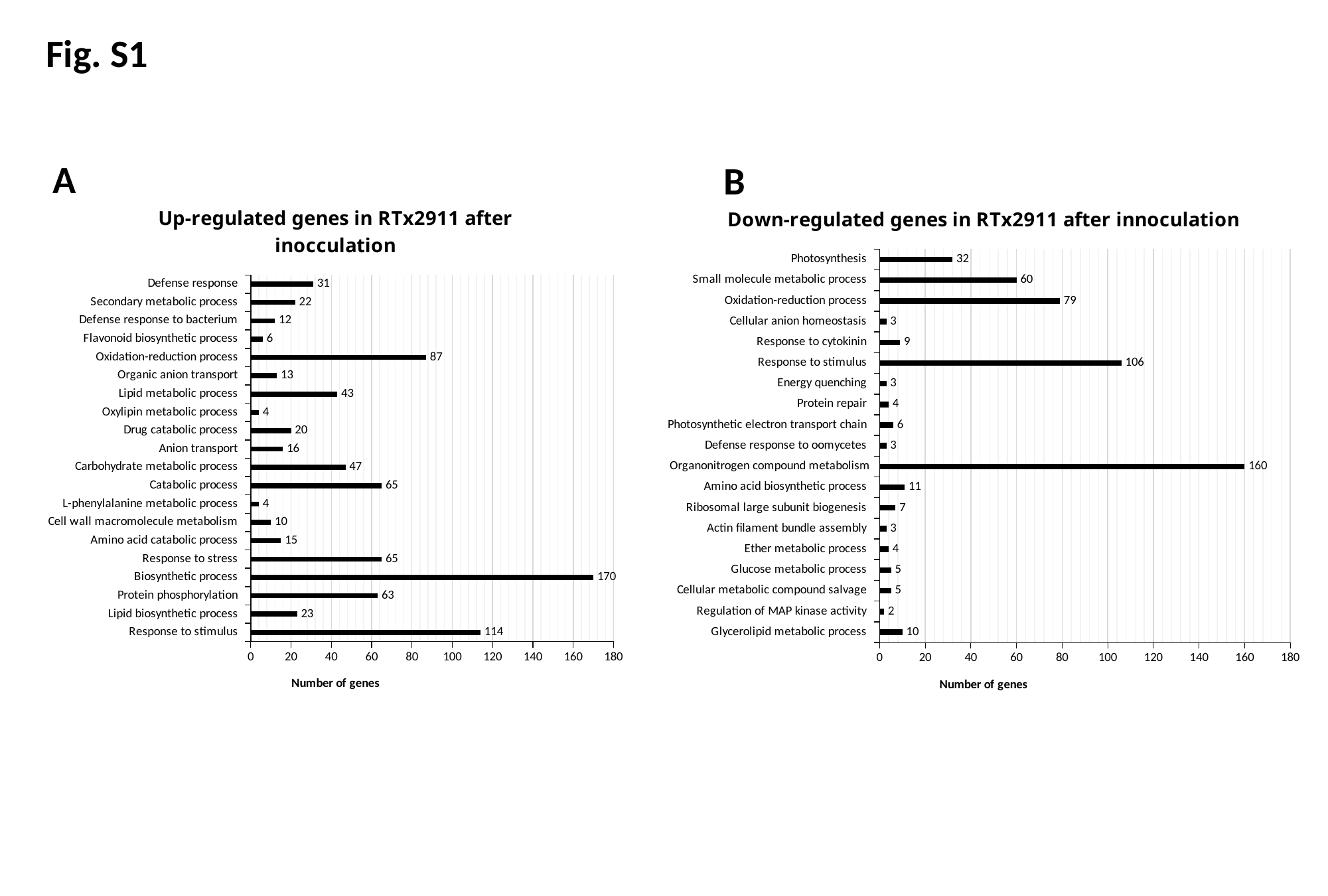

Fig. S1
A
B
### Chart: Up-regulated genes in RTx2911 after inocculation
| Category | |
|---|---|
| Response to stimulus | 114.0 |
| Lipid biosynthetic process | 23.0 |
| Protein phosphorylation | 63.0 |
| Biosynthetic process | 170.0 |
| Response to stress | 65.0 |
| Amino acid catabolic process | 15.0 |
| Cell wall macromolecule metabolism | 10.0 |
| L-phenylalanine metabolic process | 4.0 |
| Catabolic process | 65.0 |
| Carbohydrate metabolic process | 47.0 |
| Anion transport | 16.0 |
| Drug catabolic process | 20.0 |
| Oxylipin metabolic process | 4.0 |
| Lipid metabolic process | 43.0 |
| Organic anion transport | 13.0 |
| Oxidation-reduction process | 87.0 |
| Flavonoid biosynthetic process | 6.0 |
| Defense response to bacterium | 12.0 |
| Secondary metabolic process | 22.0 |
| Defense response | 31.0 |
### Chart: Down-regulated genes in RTx2911 after innoculation
| Category | |
|---|---|
| Glycerolipid metabolic process | 10.0 |
| Regulation of MAP kinase activity | 2.0 |
| Cellular metabolic compound salvage | 5.0 |
| Glucose metabolic process | 5.0 |
| Ether metabolic process | 4.0 |
| Actin filament bundle assembly | 3.0 |
| Ribosomal large subunit biogenesis | 7.0 |
| Amino acid biosynthetic process | 11.0 |
| Organonitrogen compound metabolism | 160.0 |
| Defense response to oomycetes | 3.0 |
| Photosynthetic electron transport chain | 6.0 |
| Protein repair | 4.0 |
| Energy quenching | 3.0 |
| Response to stimulus | 106.0 |
| Response to cytokinin | 9.0 |
| Cellular anion homeostasis | 3.0 |
| Oxidation-reduction process | 79.0 |
| Small molecule metabolic process | 60.0 |
| Photosynthesis | 32.0 |
