# Supplementary material for: Transcriptome analysis of early stages of sorghum grain mold disease reveals defense regulators and metabolic pathways associated with resistance
Source: BMC Genomics. 2021 Apr 22;22:295. doi: 10.1186/s12864-021-07609-y (PMC8063297; doi:10.1186/s12864-021-07609-y)
Supplement: Supplementary file 2 — Additional file 2 Fig. S2. Gene Ontology enrichment analysis of DEGs between RTx2911 and RTx430 at 24 hpi. a Enriched GO molecular process of up-regulated genes at 24 hpi in RTx2911 compared to RTx430. b Enriched GO molecular process of down-regulated genes at 24 hpi in RTx2911 compared to RTx430. [file 12864_2021_7609_MOESM2_ESM.pptx]

## Slide 1
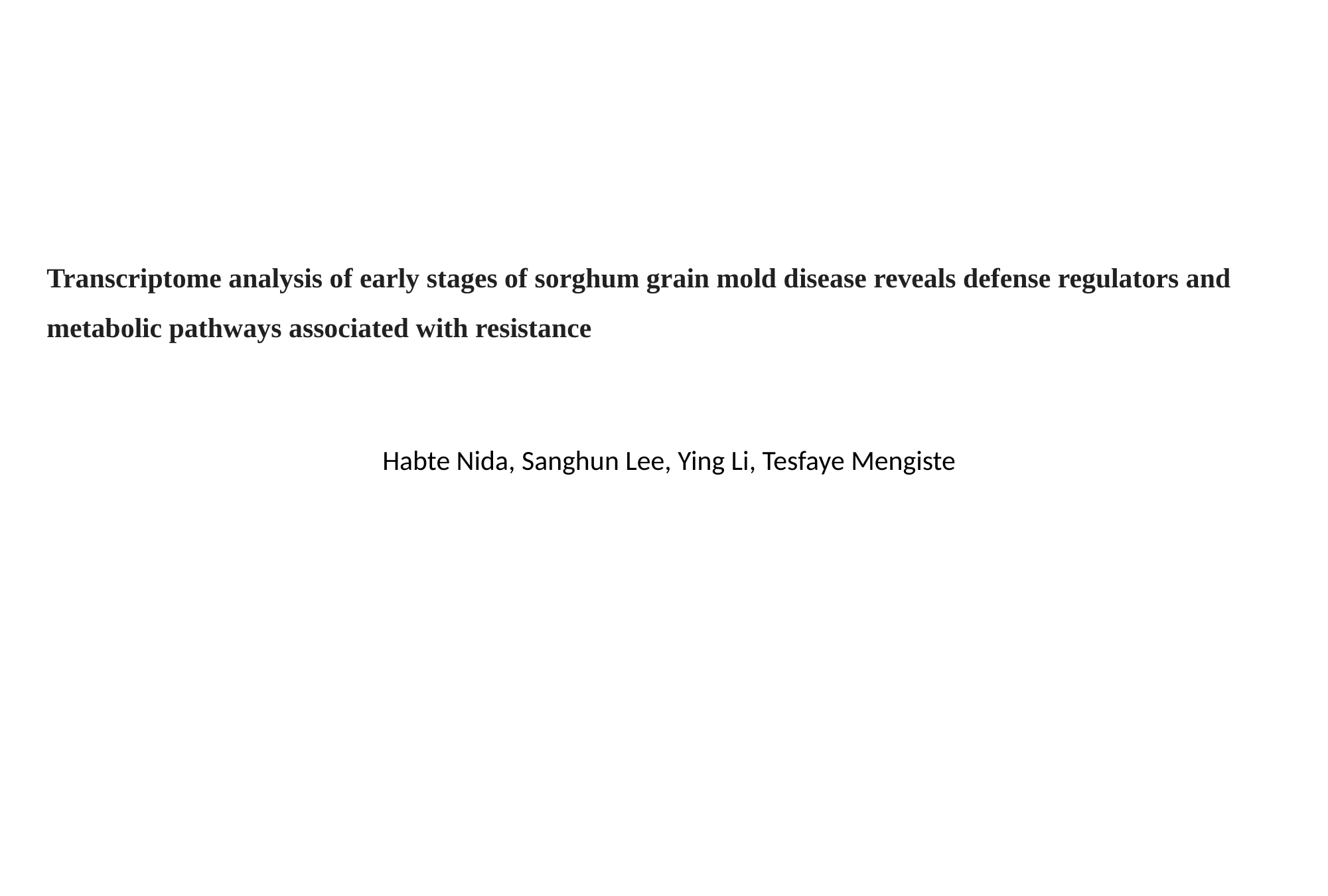

Transcriptome analysis of early stages of sorghum grain mold disease reveals defense regulators and metabolic pathways associated with resistance
Habte Nida, Sanghun Lee, Ying Li, Tesfaye Mengiste

## Slide 2
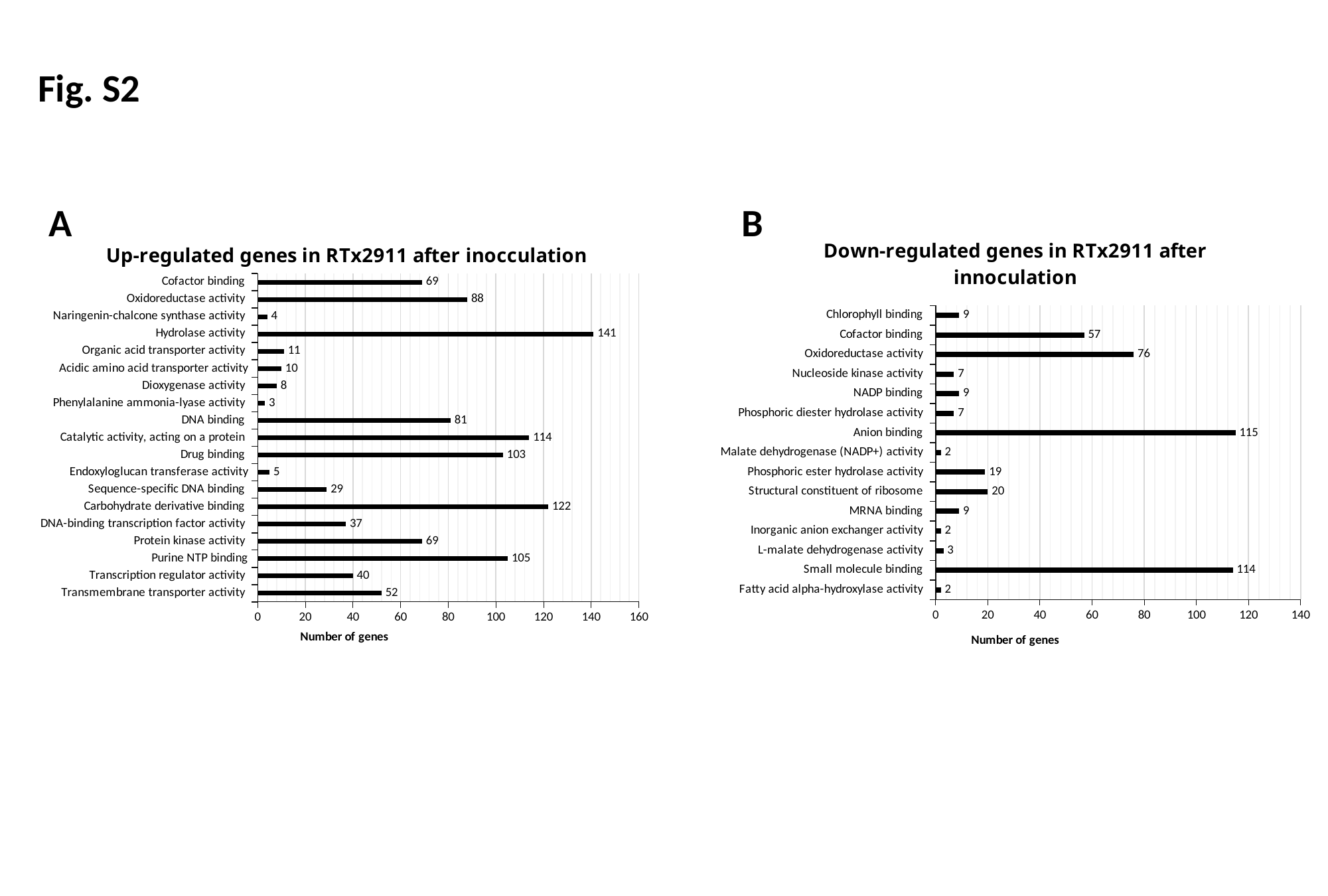

Fig. S2
A
B
### Chart: Down-regulated genes in RTx2911 after innoculation
| Category | |
|---|---|
| Fatty acid alpha-hydroxylase activity | 2.0 |
| Small molecule binding | 114.0 |
| L-malate dehydrogenase activity | 3.0 |
| Inorganic anion exchanger activity | 2.0 |
| MRNA binding | 9.0 |
| Structural constituent of ribosome | 20.0 |
| Phosphoric ester hydrolase activity | 19.0 |
| Malate dehydrogenase (NADP+) activity | 2.0 |
| Anion binding | 115.0 |
| Phosphoric diester hydrolase activity | 7.0 |
| NADP binding | 9.0 |
| Nucleoside kinase activity | 7.0 |
| Oxidoreductase activity | 76.0 |
| Cofactor binding | 57.0 |
| Chlorophyll binding | 9.0 |
### Chart: Up-regulated genes in RTx2911 after inocculation
| Category | |
|---|---|
| Transmembrane transporter activity | 52.0 |
| Transcription regulator activity | 40.0 |
| Purine NTP binding | 105.0 |
| Protein kinase activity | 69.0 |
| DNA-binding transcription factor activity | 37.0 |
| Carbohydrate derivative binding | 122.0 |
| Sequence-specific DNA binding | 29.0 |
| Endoxyloglucan transferase activity | 5.0 |
| Drug binding | 103.0 |
| Catalytic activity, acting on a protein | 114.0 |
| DNA binding | 81.0 |
| Phenylalanine ammonia-lyase activity | 3.0 |
| Dioxygenase activity | 8.0 |
| Acidic amino acid transporter activity | 10.0 |
| Organic acid transporter activity | 11.0 |
| Hydrolase activity | 141.0 |
| Naringenin-chalcone synthase activity | 4.0 |
| Oxidoreductase activity | 88.0 |
| Cofactor binding | 69.0 |
