# Supplementary material for: Transcriptome analysis of early stages of sorghum grain mold disease reveals defense regulators and metabolic pathways associated with resistance
Source: BMC Genomics. 2021 Apr 22;22:295. doi: 10.1186/s12864-021-07609-y (PMC8063297; doi:10.1186/s12864-021-07609-y)
Supplement: Supplementary file 3 — Additional file 3 Fig. S3. Enriched GO biological processes between 0 and 24 hpi for RTx2911 and RTx430. a Up-regulated genes at 24 hpi in RTx2911 compared to 0 hpi. b Up-regulated genes at 24 hpi in RTx430 compared to 0 hpi. c Down-regulated genes at 24 hpi in RTx2911 compared to 0 hpi. d Down-regulated genes at 24 hpi in RTx2911 compared to 0 hpi. [file 12864_2021_7609_MOESM3_ESM.pptx]

## Slide 1
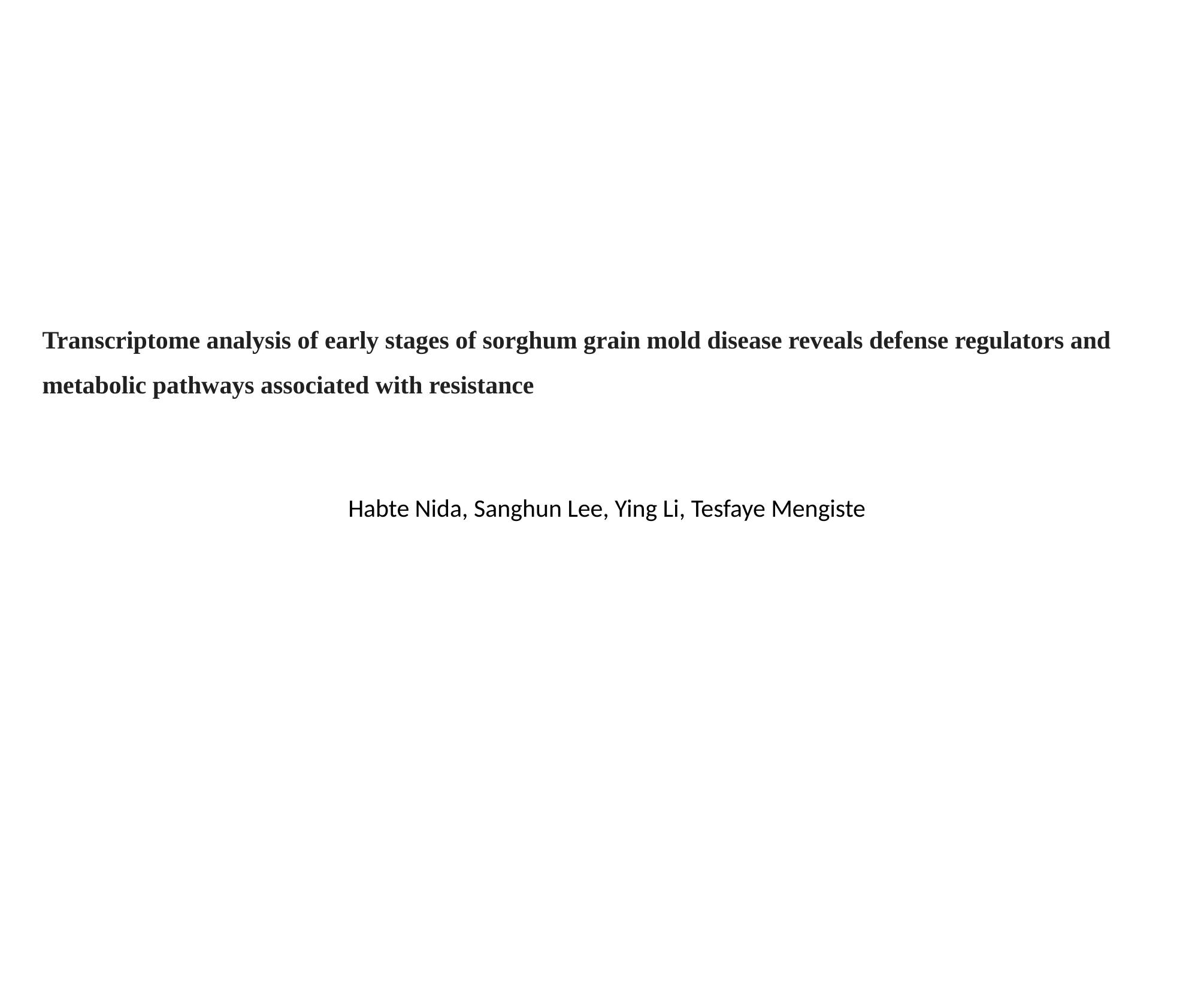

Transcriptome analysis of early stages of sorghum grain mold disease reveals defense regulators and metabolic pathways associated with resistance
Habte Nida, Sanghun Lee, Ying Li, Tesfaye Mengiste

## Slide 2
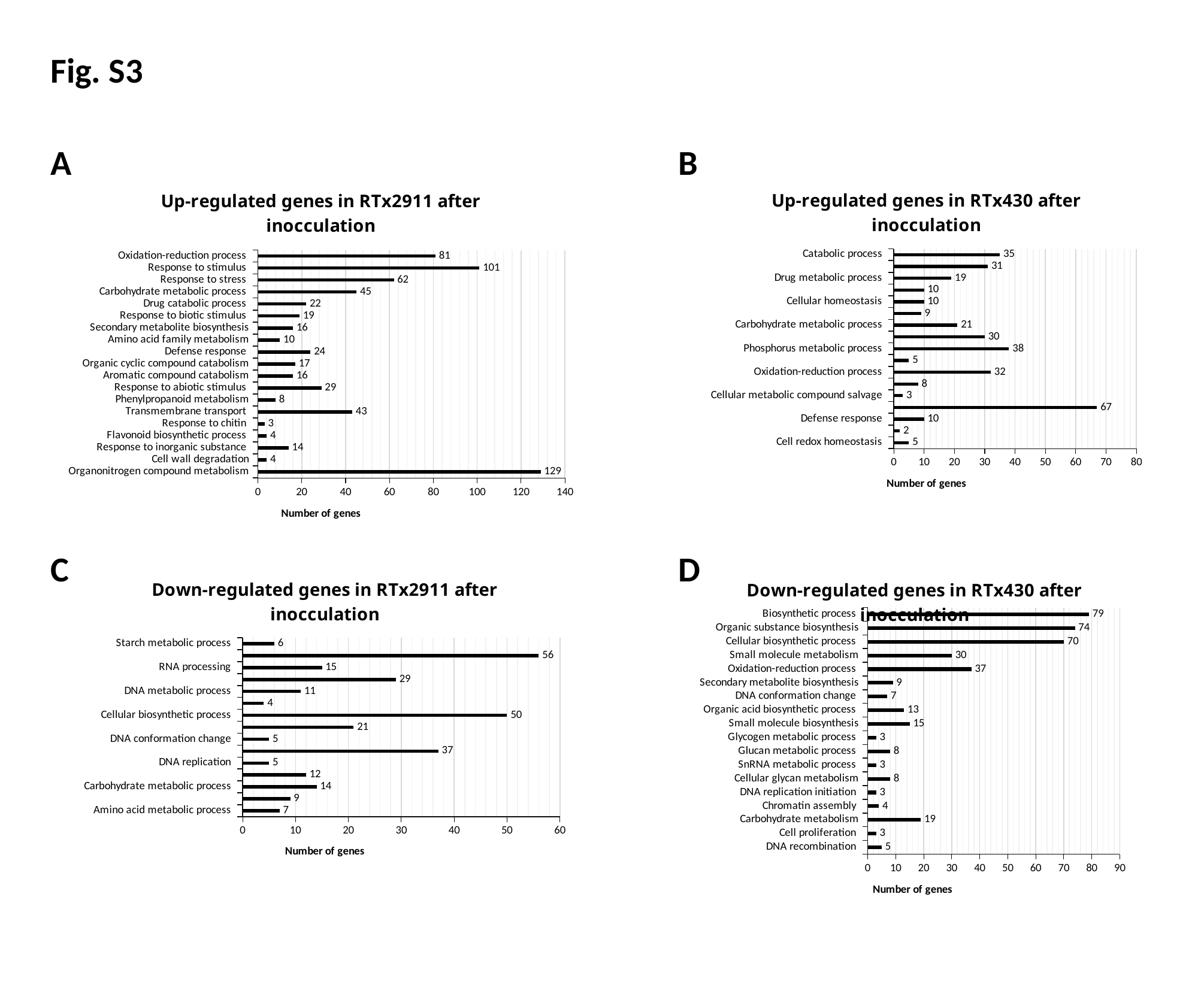

Fig. S3
A
B
### Chart: Up-regulated genes in RTx2911 after inocculation
| Category | |
|---|---|
| Organonitrogen compound metabolism | 129.0 |
| Cell wall degradation | 4.0 |
| Response to inorganic substance | 14.0 |
| Flavonoid biosynthetic process | 4.0 |
| Response to chitin | 3.0 |
| Transmembrane transport | 43.0 |
| Phenylpropanoid metabolism | 8.0 |
| Response to abiotic stimulus | 29.0 |
| Aromatic compound catabolism | 16.0 |
| Organic cyclic compound catabolism | 17.0 |
| Defense response | 24.0 |
| Amino acid family metabolism | 10.0 |
| Secondary metabolite biosynthesis | 16.0 |
| Response to biotic stimulus | 19.0 |
| Drug catabolic process | 22.0 |
| Carbohydrate metabolic process | 45.0 |
| Response to stress | 62.0 |
| Response to stimulus | 101.0 |
| Oxidation-reduction process | 81.0 |
### Chart: Up-regulated genes in RTx430 after inocculation
| Category | |
|---|---|
| Cell redox homeostasis | 5.0 |
| Regulation of carbohydrate metabolism | 2.0 |
| Defense response | 10.0 |
| Organonitrogen compound metabolism | 67.0 |
| Cellular metabolic compound salvage | 3.0 |
| Secondary metabolic process | 8.0 |
| Oxidation-reduction process | 32.0 |
| Inhibition of hydrolase activity | 5.0 |
| Phosphorus metabolic process | 38.0 |
| Phosphorylation | 30.0 |
| Carbohydrate metabolic process | 21.0 |
| Response to biotic stimulus | 9.0 |
| Cellular homeostasis | 10.0 |
| Amino acid metabolic process | 10.0 |
| Drug metabolic process | 19.0 |
| Small molecule metabolism | 31.0 |
| Catabolic process | 35.0 |C
D
### Chart: Down-regulated genes in RTx2911 after inocculation
| Category | |
|---|---|
| Amino acid metabolic process | 7.0 |
| Small molecule biosynthesis | 9.0 |
| Carbohydrate metabolic process | 14.0 |
| Carboxylic acid metabolic process | 12.0 |
| DNA replication | 5.0 |
| Gene expression | 37.0 |
| DNA conformation change | 5.0 |
| Organonitrogen compound biosynthesis | 21.0 |
| Cellular biosynthetic process | 50.0 |
| Maturation of SSU-rRNA | 4.0 |
| DNA metabolic process | 11.0 |
| Cellular component biogenesis | 29.0 |
| RNA processing | 15.0 |
| Cellular nitrogen compound metabolism | 56.0 |
| Starch metabolic process | 6.0 |
### Chart: Down-regulated genes in RTx430 after inocculation
| Category | |
|---|---|
| DNA recombination | 5.0 |
| Cell proliferation | 3.0 |
| Carbohydrate metabolism | 19.0 |
| Chromatin assembly | 4.0 |
| DNA replication initiation | 3.0 |
| Cellular glycan metabolism | 8.0 |
| SnRNA metabolic process | 3.0 |
| Glucan metabolic process | 8.0 |
| Glycogen metabolic process | 3.0 |
| Small molecule biosynthesis | 15.0 |
| Organic acid biosynthetic process | 13.0 |
| DNA conformation change | 7.0 |
| Secondary metabolite biosynthesis | 9.0 |
| Oxidation-reduction process | 37.0 |
| Small molecule metabolism | 30.0 |
| Cellular biosynthetic process | 70.0 |
| Organic substance biosynthesis | 74.0 |
| Biosynthetic process | 79.0 |
