# Supplementary material for: Transcriptome analysis of early stages of sorghum grain mold disease reveals defense regulators and metabolic pathways associated with resistance
Source: BMC Genomics. 2021 Apr 22;22:295. doi: 10.1186/s12864-021-07609-y (PMC8063297; doi:10.1186/s12864-021-07609-y)
Supplement: Supplementary file 10 — Additional file 10. Details of the workflow and python scripts used to conduct differential gene expression analysis [file 12864_2021_7609_MOESM10_ESM.docx]

**Transcriptome analysis of early stages of sorghum grain mold disease reveals defense regulators and metabolic pathways associated with resistance**

**Authors**

Habte Nida, Sanghun Lee, Ying Li, Tesfaye Mengiste

**Supplemental method**

**Details of the workflow and python scripts used to conduct differential gene expression analysis**

**Input files**

Fastq read files:

- RTx430T0Rep1_R1-filtered.fastq.gz
- RTx430T0Rep1_R2-filtered.fastq.gz
- RTx430T0Rep2_R1-filtered.fastq.gz
- RTx430T0Rep2_R2-filtered.fastq.gz
- RTx430T0Rep3_R1-filtered.fastq.gz
- RTx430T0Rep3_R2-filtered.fastq.gz
- RTx2911T0Rep1_R1-filtered.fastq.gz
- RTx2911T0Rep1_R2-filtered.fastq.gz
- RTx2911T0Rep2_R1-filtered.fastq.gz
- RTx2911T0Rep2_R2-filtered.fastq.gz
- RTx2911T0Rep3_R1-filtered.fastq.gz
- RTx2911T0Rep3_R2-filtered.fastq.gz
- RTx430T24Rep1_R1-filtered.fastq.gz
- RTx430T24Rep1_R2-filtered.fastq.gz
- RTx430T24Rep2_R1-filtered.fastq.gz
- RTx430T24Rep2_R2-filtered.fastq.gz
- RTx430T24Rep3_R1-filtered.fastq.gz
- RTx430T24Rep3_R2-filtered.fastq.gz
- RTx2911T24Rep1_R1-filtered.fastq.gz
- RTx2911T24Rep1_R2-filtered.fastq.gz
- RTx2911T24Rep2_R1-filtered.fastq.gz
- RTx2911T24Rep2_R2-filtered.fastq.gz
- RTx2911T24Rep3_R1-filtered.fastq.gz
- RTx2911T24Rep3_R2-filtered.fastq.gz

Reference genome and gff file:

- Sbicolor_454_v3.0.1.fa
- Sbicolor_454_v3.1.1.gene.gff3

**Workflow**

- Indexing the reference genome
- Aligning (mapping) read files to the reference using HISAT2 module
- Generate bam files for cufflinks analysis
- Assemble expressed genes and transcripts using Cufflinks analysis
- Create a file called assemblies.txt that lists the assembly file (simply copy paste the path to each transcript.gtf files in nano and save that as a single txt file
- Merging Cufflinks output with Cuffmerge to combine annotation files – this generates merged.gtf file in merged_asm folder. ‘merged.gtf file was copied to main working folder for the remaining step. Alternatively, it can be specified by defining its specific path.
- Quantifying gene/transcript expression using Cuffquant analysis – this generates abundances.cxb files for each sample in output folder. The abundances.cxb files were renamed with combining each sample name and the extension .cxb and copied to main working directory.
- Identifying DGE using cuffdiff. This is the last step to determine differential gene expression. Cuffdiff uses .cxb file generated from previous step and statistically compare genes. After this step is to explore differential analysis results using CummeRbund package in R. Sample of the analysis is as follows. List of differentially expressed genes can be retried and saved into tables (.txt format) as described by (Ref) and then can be converted to excel or other formats for GO Term and other downstream analysis.
  - $ module load bioinfo
  - $ module load r
  - $ R
  - > library(cummeRbund)
  - > cuff_Data <- readCufflinks('Cuffdiff') #this creates database
  - > csDensity(genes(cuff_Data))
  - > csScatter(genes(cuff_Data), 'RTx2911T24', 'RTx430T24')
  - > csVolcano(genes(cuff_Data), 'RTx2911T24', 'RTx430T24')

**Python scripts for each activity**

Building index of the sorghum reference genome

#!/usr/bin/python

import os

import subprocess

N = "Index2_PBS.sh"

outFile=open(N,'w')

outFile.write("#PBS -l nodes=1:ppn=2,walltime=2:00:00\n")

outFile.write("#PBS -M youremail\n") */example: outFile.write("#PBS -M hchikssa@purdue.edu\n")/

outFile.write("#PBS -m ae\n")

outFile.write("module load bioinfo\n")

outFile.write("module load hisat2/2.1.0\n")

outFile.write("cd yourdirectory\n") */example: outFile.write("cd /scratch/snyder/h/hchikssa/RNASeq/IndexPy \n")

Ind2 = "hisat2-build Sbicolor_454_v3.0.1.fa Sbindex " + "\n"

outFile.write(Ind2)

outFile.close()

cmd="qsub "+N

subprocess.call(cmd,shell=True)

Aligning using HISAT2 – Job file

#!/usr/bin/python

import os

import subprocess

cmd = 'ls *fastq.gz'

p=subprocess.Popen(cmd,shell=True,stdout=subprocess.PIPE,stderr=subprocess.STDOUT)

for file in p.stdout:

subscript = file.split('_')[1]

if subscript == "R2":

continue

ID = file.split('_')[0]

R1 = ID + "_R1-filtered.fastq.gz"

R2 = ID + "_R2-filtered.fastq.gz"

N = ID + "_hisat_PBS.sh"

outFile=open(N,'w')

outFile.write("#PBS -l nodes=1:ppn=2,walltime=2:00:00\n")

outFile.write("#PBS -M youremail\n")

outFile.write("#PBS -m ae\n")

outFile.write("module load bioinfo\n")

outFile.write("module load hisat2/2.1.0\n")

outFile.write("module load samtools\n")

outFile.write("module load bowtie\n")

outFile.write("cd yourdirectory\n")

R1 = ID + "_R1-filtered.fastq.gz"

R2 = ID + "_R2-filtered.fastq.gz"

SM = ID + ".sam"

HI = "hisat2 -p 2 --dta-cufflinks -x Sbindex --rna-strandness RF "+ "-1 "+ R1 + " -2 "+ R2 + " -S "+ SM + "\n"

outFile.write(HI)

BM = ID + ".bam"

St = "samtools sort -o "+ BM +" " + SM + "\n"

outFile.write(St)

outFile.close()

newcmd="qsub "+N

subprocess.call(newcmd,shell=True)

#python script to generate Bam files

#!/usr/bin/python

import os

import subprocess

cmd = 'ls *.sam'

p=subprocess.Popen(cmd,shell=True,stdout=subprocess.PIPE,stderr=subprocess.STDOUT)

for samfile in p.stdout:

ID = samfile.split('.')[0]

N = ID + "_SamtoBam_PBS.sh"

outFile=open(N,'w')

outFile.write("#PBS -l nodes=1:ppn=2,walltime=2:00:00\n")

outFile.write("#PBS -M youremail\n")

outFile.write("#PBS -m ae\n")

outFile.write("module load bioinfo\n")

outFile.write("module load samtools\n")

outFile.write("cd yourdirectory\n")

SM = ID + ".sam"

BM = ID + ".bam"

St = "samtools view -o "+ BM +" " + SM + "\n"

outFile.write(St)

outFile.close()

newcmd="qsub "+N

subprocess.call(newcmd,shell=True)

#python script to do cufflinks on the bam files

#!/usr/bin/python

import os

import subprocess

cmd = 'ls *.bam'

p=subprocess.Popen(cmd,shell=True,stdout=subprocess.PIPE,stderr=subprocess.STDOUT)

for bamfile in p.stdout:

ID = bamfile.split('.')[0]

N = ID + "_cufflinks_PBS.sh"

outFile=open(N,'w')

outFile.write("#PBS -l nodes=1:ppn=2,walltime=2:00:00\n")

outFile.write("#PBS -M youremail\n")

outFile.write("#PBS -m ae\n")

outFile.write("module load bioinfo\n")

outFile.write("module load cufflinks\n")

outFile.write("cd yourdirectory\n")

BM = ID + ".bam"

CF = "Cufflinks" +"-"+ ID

cfl = "cufflinks -G Sbicolor_454_v3.1.1.gene.gff3 -o "+ CF +" " + BM + "\n"

outFile.write(cfl)

outFile.close()

newcmd="qsub "+N

subprocess.call(newcmd,shell=True)

cuffmerge

#!/usr/bin/python

import os

import subprocess

N = "Cuffmerge_PBS.sh"

outFile=open(N,'w')

outFile.write("#PBS -l nodes=1:ppn=2,walltime=2:00:00\n")

outFile.write("#PBS -M youremail\n")

outFile.write("#PBS -m ae\n")

outFile.write("module load bioinfo\n")

outFile.write("module load samtools/1.7\n")

outFile.write("module load cufflinks/2.2.1\n")

outFile.write("cd yourdirectory\n")

CFM = "cuffmerge -g Sbicolor_454_v3.1.1.gene.gff3 -s Sbicolor_454_v3.0.1.fa assemblies.txt " + "\n"

outFile.write(CFM)

outFile.close()

cmd="qsub "+N

subprocess.call(cmd,shell=True)

#Note: assemblies.txt file was created by listing each of the .gtf files generated after cufflinks in text editor (nano):

/scratch/snyder/h/hchikssa/RNASeq/Cufflinks2-RTx430T0Rep1/RTx430T0Rep1.gtf

/scratch/snyder/h/hchikssa/RNASeq/Cufflinks2-RTx430T0Rep2/RTx430T0Rep2.gtf

/scratch/snyder/h/hchikssa/RNASeq/Cufflinks2-RTx430T0Rep3/RTx430T0Rep3.gtf

/scratch/snyder/h/hchikssa/RNASeq/Cufflinks2-RTx430T24Rep1/RTx430T24Rep1.gtf

/scratch/snyder/h/hchikssa/RNASeq/Cufflinks2-RTx430T24Rep2/RTx430T24Rep2.gtf

/scratch/snyder/h/hchikssa/RNASeq/Cufflinks2-RTx430T24Rep3/RTx430T24Rep3.gtf

/scratch/snyder/h/hchikssa/RNASeq/Cufflinks2-RTx2911T0Rep1/RTx2911T0Rep1.gtf

/scratch/snyder/h/hchikssa/RNASeq/Cufflinks2-RTx2911T0Rep2/RTx2911T0Rep2.gtf

/scratch/snyder/h/hchikssa/RNASeq/Cufflinks2-RTx2911T0Rep3/RTx2911T0Rep3.gtf

/scratch/snyder/h/hchikssa/RNASeq/Cufflinks2-RTx2911T24Rep1/RTx2911T24Rep1.gtf

/scratch/snyder/h/hchikssa/RNASeq/Cufflinks2-RTx2911T24Rep2/RTx2911T24Rep2.gtf

/scratch/snyder/h/hchikssa/RNASeq/Cufflinks2-RTx2911T24Rep3/RTx2911T24Rep3.gtf

#python script for cuffquant analysis

#!/usr/bin/python

import os

import subprocess

cmd = 'ls *.bam'

p=subprocess.Popen(cmd,shell=True,stdout=subprocess.PIPE,stderr=subprocess.STDOUT)

for bamfile in p.stdout:

ID = bamfile.split('.')[0]

N = ID + "_cuffquant_PBS.sh"

outFile=open(N,'w')

outFile.write("#PBS -l nodes=1:ppn=2,walltime=2:00:00\n")

outFile.write("#PBS -M youremail\n")

outFile.write("#PBS -m ae\n")

outFile.write("module load bioinfo\n")

outFile.write("module load cufflinks\n")

outFile.write("cd yourdirectory\n")

BM = ID + ".bam"

CQ = "./Cuffquant2" +"-"+ ID

cfq = "cuffquant -o "+ CQ +" merged.gtf "+ BM + "\n"

outFile.write(cfq)

outFile.close()

newcmd="qsub "+N

subprocess.call(newcmd,shell=True)

Cuffdiff

#!/usr/bin/python

import os

import subprocess

N = "Cuffdiff_PBS.sh"

outFile=open(N,'w')

outFile.write("#PBS -l nodes=1:ppn=2,walltime=2:00:00\n")

outFile.write("#PBS -M youremail\n")

outFile.write("#PBS -m ae\n")

outFile.write("module load bioinfo\n")

outFile.write("module load samtools/1.7\n")

outFile.write("module load cufflinks/2.2.1\n")

outFile.write("cd yourdirectory\n")

CFD = " cuffdiff -o ./Cuffdiff_24hr -v merged.gtf -L RTx2911T24,RTx430T24 RTx2911T24Rep1.cxb,RTx2911T24Rep2.cxb,RTx2911T24Rep3.cxb RTx430T24Rep1.cxb,RTx430T24Rep2.cxb,RTx430T24Rep3.cxb " + "\n"

outFile.write(CFD)

outFile.close()

cmd="qsub "+N

subprocess.call(cmd,shell=True)
